# Integrative multiomics analysis reveals the ameliorative effects of Xiasangju on metabolic dysfunction-associated steatohepatitis

**DOI:** 10.1186/s13020-025-01275-y

**Published:** 2026-01-04

**Authors:** Feng Xiang, Zhiqiang He, Chen Yang, Limei Lin, Qinghua Peng, Zhimin Zhang

**Affiliations:** 1 Key Laboratory for Quality Evaluation of Bulk Herbs of Hunan Province, School of Pharmacy, Hunan University of Chinese Medicine, Changsha, 410208 Hunan China; 2 Hunan University of Chinese Medicine, 300 Bachelor’s Road, Yuelu District, Changsha, 410208 Hunan China

**Keywords:** Xiasangju, MASH, CDAHFD, Liver fibrosis, Multiomics

## Abstract

**Background:**

Metabolic dysfunction-associated steatohepatitis (MASH) is a prevalent chronic liver disease for which safe and effective therapeutic options remain scarce. Xiasangju (XSJ), a widely consumed traditional Chinese herbal tea, exhibits diverse pharmacological activities, such as antioxidant, anti-inflammatory, and glucolipid-metabolic regulatory activities. However, its therapeutic potential for MASH has yet to be systematically explored.

**Purpose:**

This study aims to investigate the pharmacological effects of XSJ on a MASH model induced by a choline-deficient, L-amino acid-defined, high-fat diet (CDAHFD) in mice and to elucidate its potential mechanisms of action.

**Methods:**

The chemical constituents of XSJ were identified using UPLC-Q-TOF–MS technology. Network pharmacology was employed to predict the potential mechanisms of XSJ in the treatment of MASH. The therapeutic efficacy was evaluated using a CDAHFD-induced mouse model of MASH. Untargeted metabolomics and transcriptomics were utilized to elucidate key regulatory pathways, while RT-qPCR, Western blotting, and molecular docking were used to validate the underlying mechanisms.

**Results:**

A total of 74 chemical constituents in XSJ were identified by UPLC-Q-TOF–MS, predominantly phenolic acids and flavonoids. XSJ ameliorated liver injury, lipid deposition, inflammation, oxidative stress, and liver fibrosis in MASH mice. Metabolomic analysis revealed that XSJ could modulate key metabolic pathways, including purine metabolism, arginine biosynthesis, retinol metabolism, and pantothenate and CoA biosynthesis, thereby alleviating liver metabolic dysfunction. Transcriptomic analysis further revealed the regulatory effect of XSJ on the expression of genes related to cholesterol biosynthesis and metabolism, inflammation, and fibrosis. Additionally, XSJ suppressed the progression of liver fibrosis by inhibiting the TGF-β1/Smads and PI3K/AKT/Hmox1 signaling pathways.

**Conclusion:**

The findings of this study support the potential of XSJ as a therapeutic agent for MASH, revealing its synergistic mechanisms involving multiple components, targets, and signaling pathways. These results offer valuable insights for the development of novel therapeutic strategies.

**Graphical abstract:**

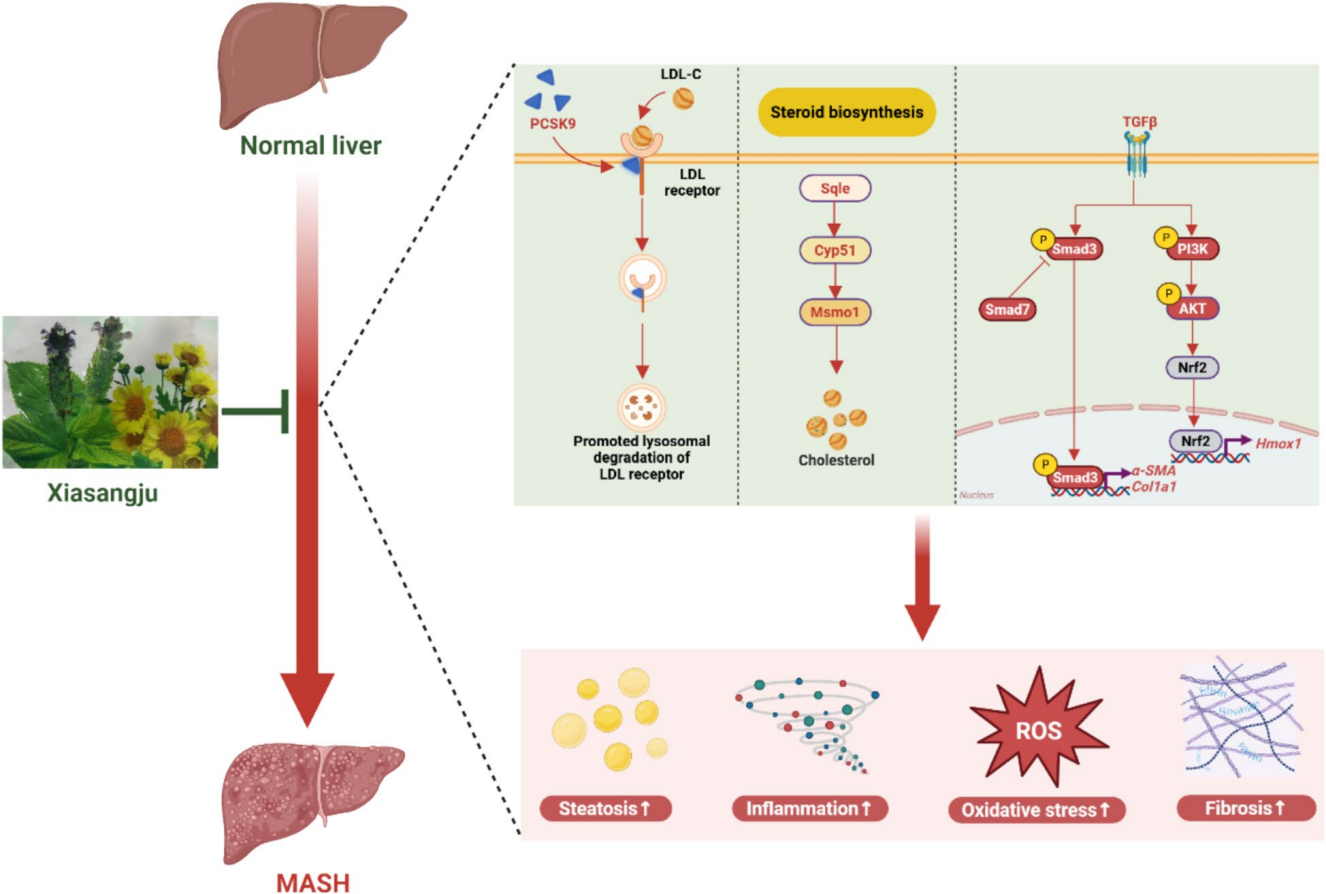

**Supplementary Information:**

The online version contains supplementary material available at 10.1186/s13020-025-01275-y.

## Highlights

- XSJ can alleviate liver damage, lipid deposition, inflammatory response, oxidative stress and liver fibrosis in the CDAHFD-induced MASH model.
- XSJ can regulate key metabolic pathways, such as purine metabolism, arginine biosynthesis, retinol metabolism, and pantothenate and CoA biosynthesis.
- XSJ can regulate cholesterol biosynthesis and metabolism, the inflammatory response and the expression of genes related to fibrosis.
- XSJ may inhibit liver fibrosis by suppressing the TGF-β1/Smads and PI3K/AKT/Hmox1 signaling pathways.

**Supplementary Information:**

The online version contains supplementary material available at 10.1186/s13020-025-01275-y.

## Introduction

Metabolic dysfunction-associated steatotic liver disease (MASLD) is a chronic liver disease closely associated with obesity, diabetes, and metabolic syndrome and affects more than 30% of adults worldwide [1]. Metabolic dysfunction-associated steatohepatitis (MASH), previously known as nonalcoholic steatohepatitis (NASH), represents an advanced form of MASLD. MASH is characterized by hepatic steatosis, inflammatory responses, and varying degrees of liver fibrosis. Liver fibrosis, a critical hallmark of advanced MASH, may progress to cirrhosis and hepatocellular carcinoma if the underlying etiology persists [2]. In recent years, the development of effective therapeutic strategies to alleviate liver fibrosis has become a major focus of research.

Liver fibrosis is a pathological process involving multiple factors and complex mechanisms. The “multiple hit” mechanism, driven by the synergistic actions of lipid accumulation, oxidative stress, and inflammatory responses, collectively drives disease progression [3]. The core pathological feature of liver fibrosis is the abnormal deposition of extracellular matrix (ECM), which is mediated primarily by activated hepatic stellate cells (HSCs). Under physiological conditions, HSCs remain in a quiescent state. However, upon exposure to repeated liver injury stimuli, these cells can be activated and transdifferentiated into myofibroblasts, characterized by the upregulation of α-smooth muscle actin (α-SMA) and collagen type I alpha 1 (Col1a1) [4]. Transforming growth factor-β1 (TGF-β1) is a key profibrotic factor in the process of liver fibrosis. It activates the mothers against decapentaplegic homolog 3 (Smad3) signaling pathway, inducing its phosphorylation and oligomerization, and subsequently enters the nucleus to regulate the expression of TGF-β1 target genes [5]. Smad7, a negative regulator of the TGF-β1/Smad3 signaling pathway, can inhibit the TGF-β1-mediated fibrotic response [6]. Modulating the TGF-β1/Smads signaling pathway is an important therapeutic strategy for inhibiting HSC activation and delaying the progression of liver fibrosis. Additionally, TGF-β1 can activate the phosphoinositide-3 kinase (PI3K)/protein kinase B (AKT) pathway in a Smad-independent manner [7]. Numerous studies have confirmed that regulating the PI3K/AKT pathway can effectively inhibit the occurrence and development of liver fibrosis [8, 9]. Further research has revealed that the PI3K/AKT pathway can promote the nuclear translocation of nuclear factor erythroid 2-related factor 2 (Nrf2), thereby upregulating the expression of heme oxygenase 1 (Hmox1/HO-1) [10, 11]. Overexpression of Hmox1 can catalyze the degradation of heme, releasing iron ions, which in turn trigger ferroptosis [12, 13], thereby exacerbating the pathological process of liver fibrosis [14]. Therefore, abnormal activation of the PI3K/AKT/Hmox1 pathway may play a critical role in the development of liver fibrosis, suggesting that it could serve as a novel therapeutic intervention target.

Traditional Chinese medicine (TCM) formulas, characterized by their multicomponent, multitarget, and multipathway properties, have demonstrated significant therapeutic potential and broad application prospects in the treatment of liver fibrosis. Xiasangju (XSJ), a classic TCM formula composed of *Prunella vulgaris* L., *Morus alba* L., and *Chrysanthemum indicum* L., originates from the prescription “Sang Ju Yin” in the book “*Detailed Analysis of Epidemic Warm Diseases* (*Wenbing Tiaobian*)” written by the renowned Qing Dynasty physician Wu Jutong. XSJ is renowned for its efficacy in clearing liver heat, improving eyesight, detoxifying, and clearing heat and serves as a refreshing beverage that is both medicinal and edible [15]. In recent years, with advances in modern pharmacological research, multiple active components in XSJ have been confirmed to have a wide range of biological effects, including antioxidant, anti-inflammatory, and metabolic regulatory activities [16–18]. These effects have shown great potential for the prevention and treatment of liver diseases. However, the potential value of XSJ in the treatment of MASH has not yet been fully explored.

In recent years, a mouse model induced by a choline-deficient, L-amino acid-defined, high-fat diet (CDAHFD) has been widely used to simulate the pathological process of MASH. This model can rapidly induce significant liver fibrosis within 6–9 weeks and has good pathological similarity and research value [19, 20]. Therefore, in this study, we employed the CDAHFD-induced MASH model for in vivo research. By integrating bioinformatics analysis and multiomics technologies, we systematically explored the therapeutic potential of XSJ in the treatment of MASH and further elucidated its possible antifibrotic mechanisms.

## Methods and materials

### Materials and reagents

XSJ granules (Chinese drug approval number Z44022217) were provided by Baiyunshan Xingqun Pharmaceutical Co., Ltd. (Guangzhou, China). C57BL/6 male mice (18–22 g, 6 weeks old) were purchased from Hunan Silex Jingda Laboratory Animal Co., Ltd. (Changsha, China). CDAHFD was purchased from Jiangsu Xietong Pharmaceutical Bioengineering Co., Ltd. (XTM07-006, Nanjing, China). Total cholesterol (A111-1–1, TC), triglycerides (A110-1–1, TG), malondialdehyde (A003-1–2, MDA), superoxide dismutase (A001-3–2, SOD), catalase (A007-1–1, CAT), and glutathione peroxidase (A005-1–2, GSH-Px) were obtained from Nanjing Jiancheng Bioengineering Institute (Nanjing, China). The mouse interleukin-1β (MM-0040M1, IL-1β) ELISA Kit, mouse interleukin-6 (MM-0163M1, IL-6) ELISA Kit, and mouse tumor necrosis factor-α (MM-0132M1, TNF-α) ELISA Kit were purchased from Jiangsu Meimian Industrial Co., Ltd. (Yancheng, China).

### UPLC-Q-TOF-MS analysis of XSJ

To identify the main components of the XSJ granules, 100 mg of XSJ was dissolved in 1 mL of methanol and subjected to ultrasonic extraction for 10 min. The solution was subsequently centrifuged at 4000 rpm for 10 min at room temperature and filtered through a 0.22 μm microporous membrane. The filtered sample was analyzed using UPLC-Q-TOF–MS (5600 + System, AB SCIEX, USA). Chromatographic separation was performed on an ACQUITY UPLC BEH C_18_ column (100 mm × 2.1 mm, 1.7 μm) at a column temperature of 40 °C. The injection volume was 5 μL, with a flow rate of 0.4 mL/min. The mobile phase consisted of 0.1% formic acid in water (A) and 0.1% formic acid in acetonitrile (B). Mass spectrometric analysis was carried out in negative ion mode, with a mass range of *m/z* 100 to 1200. Tandem mass spectrometry (MS/MS) data were acquired using information-dependent acquisition (IDA).

### Network pharmacology

The potential targets of the components identified by UPLC-Q-TOF–MS in XSJ were predicted using the SwissTargetPrediction database (https://swisstargetprediction.ch/). MASH-related targets were identified from the GeneCards database (https://www.genecards.org/) and the OMIM database (https://www.omim.org/) using the search terms “NASH,” “MASH,” and “Liver fibrosis.” A Venn analysis was performed to identify the overlapping targets, thereby determining the potential active targets of XSJ in the treatment of MASH. Gene Ontology (GO) and Kyoto Encyclopedia of Genes and Genomes (KEGG) enrichment analyses were subsequently conducted on these targets, and the 20 significantly enriched GO and KEGG terms are presented in graphical form. Finally, Cytoscape 3.7.1 software was used to construct a “drug-component-target-pathway-disease” network diagram, providing a comprehensive visualization of the potential molecular mechanisms underlying the therapeutic effects of XSJ on MASH.

### Design of the animal experiment

The animals were housed in a controlled environment (temperature 22–24 °C, relative humidity 60–65%, and a 12-h light cycle) and maintained under specific pathogen-free conditions. They were acclimated for 1 week prior to the experiment. The animal experiments strictly followed the international rules and ethical principles of animal use and care in the laboratory of Hunan University of Chinese Medicine (Approval No. SLBH-202409020003). This study aimed to evaluate the protective effects of XSJ on CDAHFD-induced MASH. A total of 50 mice were randomly divided into five groups. The control group (n = 10) was fed a standard diet. The model group (n = 10) was fed a CDAHFD. On the basis of the body surface area conversion ratio between mice and humans, the human adult clinical dose was converted to an equivalent dose for mice. The equivalent dose of XSJ for mice (g/kg) was calculated using the following formula: body surface area conversion ratio between mice and humans × adult daily dose/adult average body weight. That is, 9.1 × 60 (g)/70 (kg) = 7.8 g/kg as the equivalent dose for mice. For experimental design convenience, 8.0 g/kg was set as the high dose, 4.0 g/kg as the medium dose, and 2.0 g/kg as the low dose. XSJ granules were dissolved in normal saline and administered to mice by gavage at low (2.0 g/kg), medium (4.0 g/kg), and high (8.0 g/kg) doses (n = 10) daily for 6 weeks. All the mice had free access to water. After the experiment, serum and liver samples were obtained and stored at − 80 °C.

### Detection of serum and liver biochemical indicators

Blood samples were allowed to stand at room temperature for 30 min to facilitate clotting, followed by centrifugation at 3000 rpm for 5 min at 4 °C to separate the serum. The levels of alanine aminotransferase (ALT) and aspartate aminotransferase (AST) in the serum were measured using an automatic biochemical analyzer (BIOBASE, BK-280). The contents of TG and TC in liver tissue, as well as the activities of antioxidant markers such as MDA, SOD, CAT, and GSH-Px, were determined according to the instructions provided in the respective assay kits. Additionally, the expression levels of inflammatory cytokines, such as IL-1β, IL-6, and TNF-α, in the liver were quantitatively analyzed following the detection protocols outlined in the corresponding ELISA kits.

### Histological analysis

Liver tissue samples were initially fixed in a 4% paraformaldehyde solution for 24 h. The tissues subsequently underwent gradient ethanol dehydration, clearing, and paraffin embedding, followed by the preparation of paraffin sections for hematoxylin-eosin (H&E) staining to observe histological morphological changes in the liver. Another portion of the liver tissue was embedded in OCT embedding medium, frozen, and then sectioned using a cryostat. These sections were subsequently washed with 60% isopropanol and stained with Oil Red O staining solution to visualize lipid accumulation. Additionally, some liver tissue sections were subjected to Masson's trichrome staining to evaluate the extent of liver fibrosis. Experienced liver pathologists assessed the samples under double-blind conditions and calculated the NAFLD activity score (NAS) for each group. The scores for steatosis and lobular inflammation ranged from 0 to 3, whereas the scores for hepatocellular ballooning ranged from 0 to 2 [21]. The areas positive for Oil Red O staining and collagen deposition in the Masson-stained sections were quantitatively analyzed using ImageJ software (National Institutes of Health, Bethesda, MD, USA) to further evaluate the severity of liver pathology.

### Immunohistochemical staining

The liver paraffin sections were first dewaxed with xylene and then dehydrated using a graded ethanol series. Antigen retrieval was performed on the sections using antigen retrieval solution under high-temperature and high-pressure conditions. The sections were subsequently incubated in 3% hydrogen peroxide solution at room temperature for 20 min to block the activity of endogenous peroxidases, thereby avoiding interference with the subsequent staining process. The sections were then blocked with 10% goat serum at 37 °C for 30 min to block nonspecific binding sites and reduce background staining. After blocking, the excess serum was removed by gently shaking, and primary antibodies against F4/80 (Abcam, UK, ab300421, 1:500) or α-SMA (Proteintech, China, 14395-1-AP, 1:2000) were applied and incubated overnight at 4 °C. The next day, the sections were incubated with horseradish peroxidase (HRP)-conjugated secondary antibodies at 37 °C for 1 h. The sections were then washed three times with PBS for 5 min each time. DAB substrate was applied, and the sections were observed under a microscope until specific brown staining was visible. The DAB was immediately washed off with distilled water to terminate the color development reaction. Finally, the sections were counterstained with hematoxylin, differentiated in 0.5% hydrochloric acid ethanol, dehydrated through a graded ethanol series, cleared in xylene, and mounted with neutral resin. The sections were then observed and photographed under a microscope.

### Untargeted metabolomics analysis

Six samples were randomly selected from each group of mice for untargeted metabolomics analysis. Liver tissues were thawed at 4 °C, and 50 mg of each sample was accurately weighed and transferred into a homogenization tube containing two steel beads. Subsequently, 400 μL of precooled methanol–water solution (4:1, v/v) was added. The samples were homogenized at low temperature using a tissue homogenizer, thoroughly mixed, and then sonicated on ice for 20 min. Afterward, the samples were kept at − 20 °C for 1 h. The homogenates were subsequently centrifuged at 16,000 × g at 4 °C for 20 min, after which an appropriate volume of the supernatant was collected for analysis.

The analysis was performed on a SHIMADZU-LC30 ultrahigh-performance liquid chromatography (UHPLC) system coupled with an ACQUITY UPLC^®^ HSS T3 column (2.1 × 100 mm, 1.8 µm) (Waters, Milford, MA, USA). The injection volume was 16 µL, the column temperature was maintained at 40 °C, and the flow rate was set at 0.3 mL/min. Mobile phase A consisted of 0.1% formic acid in water, while mobile phase B consisted of 0.1% formic acid in acetonitrile. The gradient elution program was as follows: 0–2 min, 0% B; 2–3.3 min, linear increase from 0 to 48% B; 3.3–5.1 min, linear increase from 48 to 100% B; 5.1–7.2 min, 100% B; 7.2–7.3 min, linear decrease from 100 to 0% B; and 7.3–10 min, 0% B.

The samples were analyzed in both positive ion (+) and negative ion (−) modes using electrospray ionization (ESI). After separation by UPLC, the samples were subjected to mass spectrometry analysis using an Orbitrap Fusion Lumos Tribrid mass spectrometer (Thermo Scientific). Ionization was performed using a HESI source with the following ionization conditions: Spray Voltage: 3.8 kV (+) and 3.2 kV (−); capillary temperature: 320 °C (±); sheath gas: 40 (±); auxiliary gas: 15 (±); probe heater temperature: 350 °C (±); and S-lens RF level: 50.

The mass spectrometry acquisition settings were as follows: mass spectrometry acquisition time, 10 min; precursor ion scan range, 75–1050 m*/z*; full MS resolution: 50,000 @ *m/z* 200; automatic gain control (AGC) target: 3e^6^; maximum injection time: 50 ms. For MS/MS analysis, the top 10 most intense precursor ions were selected for subsequent MS/MS acquisition after each full scan. MS/MS resolution: 7,500 @ *m/z* 200; AGC target: 1e^5^; maximum injection time: 50 ms; activation type: higher-energy collision dissociation; isolation window: 2 m*/z*; normalized collision energy (stepped): 20, 30, 40.

The raw data underwent peak alignment, retention time correction, and peak area extraction using MS-DIAL software. Metabolite structural identification was performed by matching accurate mass numbers (mass tolerance < 10 ppm) and MS/MS (mass tolerance < 0.01 Da) against public databases such as HMDB, MassBank, and GNPS, as well as an in-house metabolite library. For the extracted data, ion peaks with more than 50% missing values within any group were excluded from subsequent statistical analysis. The peak areas of the positive and negative ion data were normalized separately by the total peak area, and the normalized data from both ion modes were integrated and processed using Python software. Differential metabolites were screened on the basis of fold change (FC > 1.5 or FC < 1/1.5) and statistical significance (p < 0.05). The processed data were subsequently subjected to partial least squares discriminant analysis (PLS-DA) and metabolic pathway enrichment analysis.

### Transcriptomic analysis

Six samples were randomly selected from each group of mice for transcriptomic analysis. Total RNA was extracted from liver tissue samples, and RNA integrity was assessed using an Agilent 2100 Bioanalyzer, while the RNA concentration was precisely measured with a NanoDrop spectrophotometer. mRNA was isolated from the total RNA using oligo-dT-coated magnetic beads, followed by fragmentation of the captured mRNA. Subsequently, first- and second-strand cDNAs were synthesized using reverse transcriptase, and the reverse transcription products underwent end repair, followed by the addition of an adenine (A) base to the 3' ends. The resulting fragments were then ligated to sequencing adapters. After purification to remove incompletely ligated products and empty adapter dimers, PCR amplification was performed using primers complementary to the adapter sequences. The sequencing library was further purified using magnetic beads. Upon completion of library construction, the library concentration was determined using a Qubit fluorometer, and the library fragment size was analyzed with an Agilent Fragment Analyzer to ensure library quality. After passing quality control checks, the libraries were subjected to paired-end 150 bp (PE150) sequencing on an Illumina NovaSeq 6000 platform. Differentially expressed genes (DEGs) were subsequently screened on the basis of fold change (FC > 1.5 or FC < 1/1.5) and statistical significance (p < 0.05). GO enrichment analysis and KEGG pathway enrichment analysis were conducted on the identified DEGs to systematically elucidate their potential functional associations across various biological processes and signaling pathways.

### RT-qPCR analysis

Total RNA was extracted from liver tissue using TRIzol reagent according to the manufacturer's instructions (Accurate Biotechnology Co., Ltd.). The purity and total RNA concentration were determined using a NanoDrop 2000 spectrophotometer (Thermo Scientific). Complementary DNA (cDNA) templates were synthesized using the EVO M-MLV RT Mix Kit. Quantitative real-time PCR (qPCR) was performed to assess mRNA levels using SYBR Green premix. The PCR conditions were as follows: an initial predenaturation step at 95 °C for 30 s, followed by 40 amplification cycles, with each cycle consisting of denaturation at 95 °C for 5 s and annealing/extension at 60 °C for 30 s. All reactions were performed in triplicate, and the data were analyzed using LightCycler analysis software based on the 2^−ΔΔCt^ method. GAPDH was used as an internal control and amplified under the same PCR conditions. The primers used are listed in Table 1.

**Table 1:** Primer sequences

Gene	%1、 Primer Forward	%1、 Primer Reverse
α-SMA	%1、 CCCTGAAGAGCATCCGACAC	%1、 CCAGAGTCCAGCACAATACCA
Col1a1	%1、 GACATGTTCAGCTTTGTGGACCTC	%1、 GGGACCCTTAGGCCATTGTGTA
Sqle	%1、 ATCGGCGTGCAATACAAGGA	%1、 CAAGCTCCGCAAAATTGGGT
Thbs1	%1、 TACCACTGCAAAAAGGACAACT	%1、 GTCATAGTCATACTGGGCTGGG
Cyp51	%1、 GCGAGGATCTGCCTCCTTTA	%1、 GAATGGTGTACCCTGCCACC
Msmo1	%1、 ATGTGGACTCACTGTTGCCG	%1、 ATGGCTTCGTGAACTATCAGGG
Pcsk9	%1、 TTATGAAGAGCTGATGCTCGC	%1、 ACAATGTAGGTTCCTGGCAGC
Timp4	%1、 GACTCTTCCCTCTGTGGTGTG	%1、 GATTCAGGCTCTCCCTCTGC
Hamp	%1、 CCATCTGCATCTTCTGCTGTAAA	%1、 GGAGGGCAGGAATAAATAATGGG
Hamp2	%1、 TCCTGTGGTATCTGTTGTGAAGAAT	%1、 GTCATTGCTGGAGAGGGCAG
Hmox1	%1、 CTGGAGATGACACCTGAGGTCAA	%1、 CTGACGAAGTGACGCCATCTG
Ticam2	%1、 TAGACGATGCGGTCAATGGG	%1、 TTGTACTTGTGCTGCCTGCT
Mthfd2	%1、 GATCCTGTCACTGCAAAGCC	%1、 ACTTCCAGCTCCTCTGGTCT
Trim29	%1、 CAAGGCCCAGCCTCAGAC	%1、 TGACATAGAATGGCCGGTAGTG
Batf	%1、 GAAAGCCGACACCCTTCACC	%1、 TGAGCTGTTTGATCTCTTTGCG
GAPDH	%1、 TGTGTCCGTCGTGGATCTGA	%1、 TTGCTGTTGAAGTCGCAGGAG

### Western blotting

100 mg of liver tissue was added to RIPA lysis buffer in a grinding tube, the sample was further homogenized using a grinder, and the sample was centrifuged at 12000 rpm for 20 min at 4 °C. The supernatant was collected, and the protein concentration was quantified using the BCA method. On the basis of the determined protein concentrations, an appropriate volume of loading buffer was added, and the samples were heated in a liquid metal bath to denature the proteins. The proteins were subsequently separated using SDS-PAGE gels of varying concentrations and transferred onto PVDF membranes. The membranes were blocked with 5% BSA for 90 min, followed by incubation with the corresponding primary antibodies at 4 °C overnight, including mouse anti-GAPDH (Proteintech, China, 60004-1-Ig, 1:5000), rabbit anti-Col1a1 (Proteintech, China, 25870-1-AP, 1:3000), rabbit anti-TGF-β1 (Selleck, China, F1624, 1:1000), mouse anti-Smad3 (Proteintech, China, 66516-1-Ig, 1:6000), rabbit anti-p-Smad3 (ABclonal, China, AP0727, 1:1000), rabbit anti-Smad7 (Proteintech, China, 25840-1-AP, 1:1000), mouse anti-PI3K (Proteintech, China, 60225-1-Ig, 1:20000), rabbit anti-p-PI3K (Affinity, China, AF3241, 1:1000), rabbit anti-AKT (ABclonal, China, A17909, 1:3000), rabbit anti-p-AKT (Cell Signaling Technology, USA, 4060S, 1:2000), and rabbit anti-Hmox1 (Proteintech, China, 10701-1-AP, 1:1000) antibodies. After three 10-min TBST washes, the membranes were incubated with specific secondary antibodies at 37 °C for 60 min. The secondary antibodies used were goat anti-mouse IgG (H + L) (Elabscience, China, E-AB-1001, 1:20000) and goat anti-rabbit IgG (H + L) (Elabscience, China, E-AB-1003, 1:20000). Finally, the specific protein bands were detected using an enhanced chemiluminescence (ECL) detection system, and chemiluminescence signals were captured using a Bio-Rad imaging system (ChemiDoc™ XRS +, Bio-Rad). The grayscale values were analyzed and statistically processed using ImageJ software.

### Molecular docking

The core components of XSJ were downloaded in 2D structure format from the PubChem database (https://pubchem.ncbi.nlm.nih.gov/), converted into mol2 format using Chem3D, and further optimized for molecular structure. The PDB files of the target proteins were obtained from the RCSB PDB database (https://www.rcsb.org/), and water molecules and heteroatoms were removed using PyMOL. Subsequently, AutoDock Tools was used to convert the optimized ligands and receptor proteins into pdbqt format, and the active site parameters were obtained. After the docking region was defined, molecular docking was performed using AutoDock Vina. Finally, the docking results were visualized using PyMOL.

### Statistical analysis

All the data are presented as the mean ± standard error of the mean (SEM). For comparisons between two groups, Student's t-test was used if the data were normally distributed and had homogeneity of variance; otherwise, the Mann-Whitney U test was used as the main nonparametric test. For comparisons among multiple groups, one-way ANOVA was used if the data were normally distributed and had homogeneity of variance; otherwise, the Kruskal-Wallis test was used as the nonparametric method for assessing intergroup differences. Statistical analyses were performed using GraphPad Prism 9.0 software.

## Results

### Identification of XSJ components

**Fig. 1:**
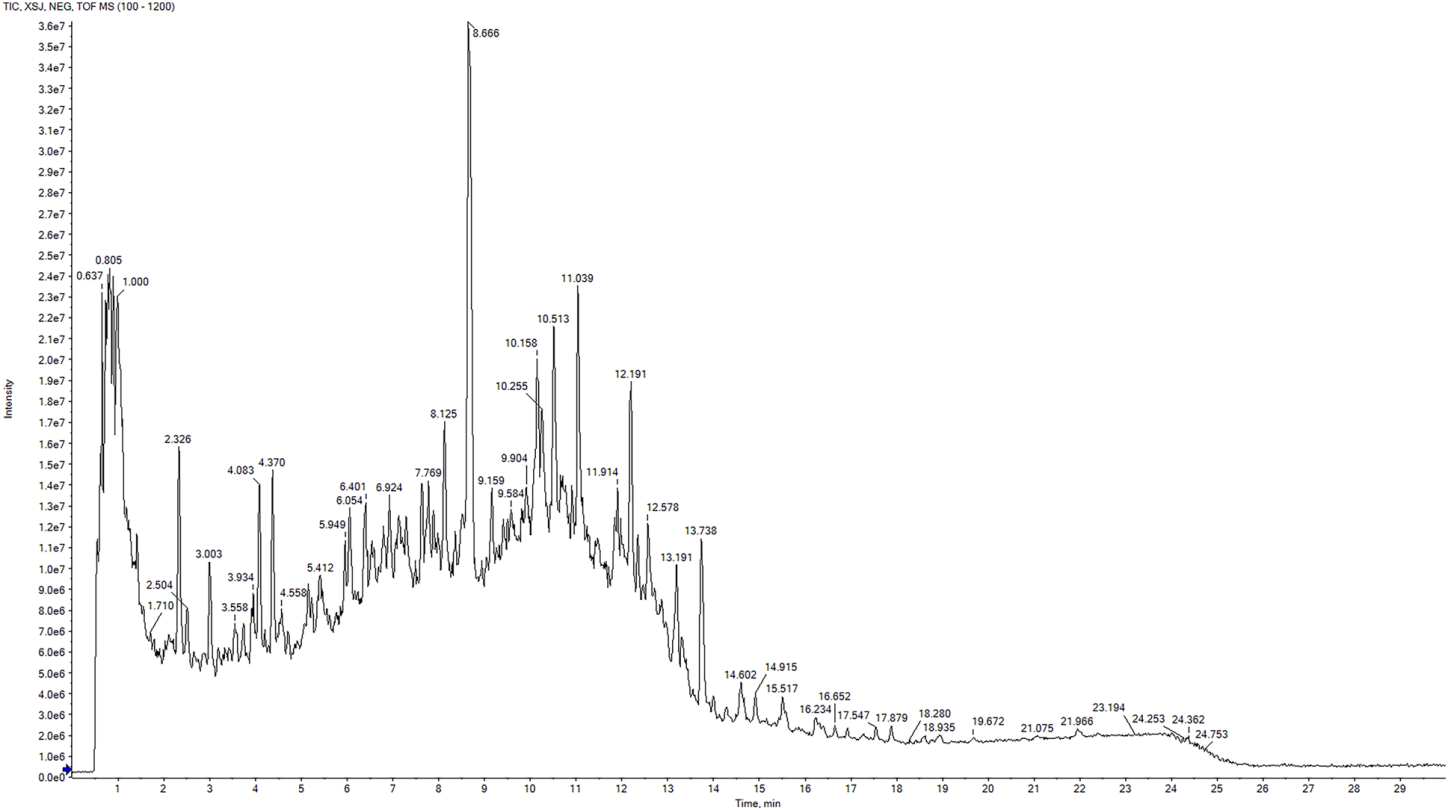
Total ion chromatogram (TIC) in negative ion mode

UPLC-Q-TOF–MS was employed to analyze the major components of XSJ. The total ion chromatogram (TIC) is presented in Fig. 1. A total of 74 chemical constituents were identified on the basis of the fragmentation patterns of standards and literature data (Supplementary file 1). The bioactive constituents of XSJ primarily originate from phenolic acids and flavonoids, which are recognized for their potential biological activities and were subsequently utilized for target and pathway prediction analyses through network pharmacology approaches.

### Network pharmacology analysis

**Fig. 2:**
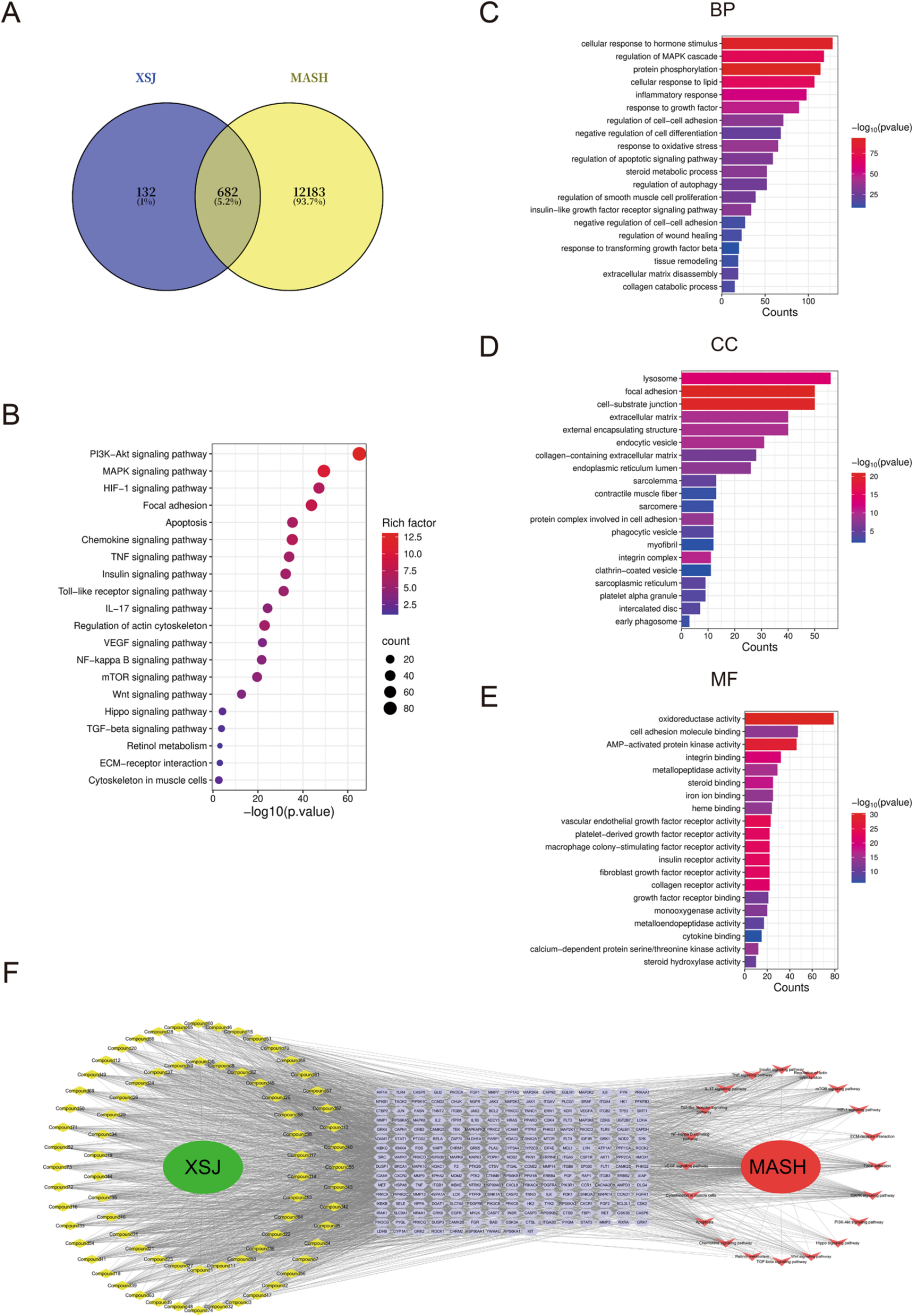
Network pharmacology prediction of the potential mechanisms of XSJ in treating MASH. **A** Venn diagram of the intersection targets of XSJ and MASH. **B** Results of the KEGG enrichment analysis. **C** Representative BP terms of core targets. **D** Representative CC terms of core targets. **E** Representative MF terms of core targets. **F** “Drug-component-target-pathway-disease” network diagram

On the basis of the 74 identified chemical constituents of XSJ, we retrieved 814 drug targets from the SwissTargetPrediction database. We subsequently searched for genes related to MASH in the GeneCards and OMIM databases, identifying a total of 12,865 disease targets. Afterward, through Venn diagram analysis, we compared the intersection of drug targets and disease targets, ultimately identifying 682 key targets for XSJ in the treatment of MASH (Fig. 2A). The results of the KEGG enrichment analysis revealed that pathways such as the PI3K-AKT signaling, chemokine signaling, TGF-β signaling, and ECM-receptor interaction pathways were significantly enriched under XSJ intervention. These pathways are closely related to the occurrence and development of liver fibrosis (Fig. 2B). Further GO gene function enrichment analysis was performed using the Metascape database (http://metascape.org/), with a significance threshold set at P < 0.05. We identified three functional categories: molecular function (MF), biological process (BP), and cellular component (CC). The results indicated that the treatment of MASH with XSJ involves a variety of biological processes, including the inflammatory response, oxidative stress, cell adhesion, cell differentiation, and extracellular matrix, all of which are strongly associated with the complex pathological processes of liver fibrosis (Fig. 2C-E). Finally, on the basis of the interactions between drug components, targets, and pathways, we constructed a “drug-component-target-pathway-disease” multidimensional network diagram, which illustrates the multicomponent, multitarget, and multipathway regulatory characteristics of the therapeutic effects of XSJ on MASH (Fig. 2F).

### XSJ ameliorates CDAHFD-induced liver injury

**Fig. 3:**
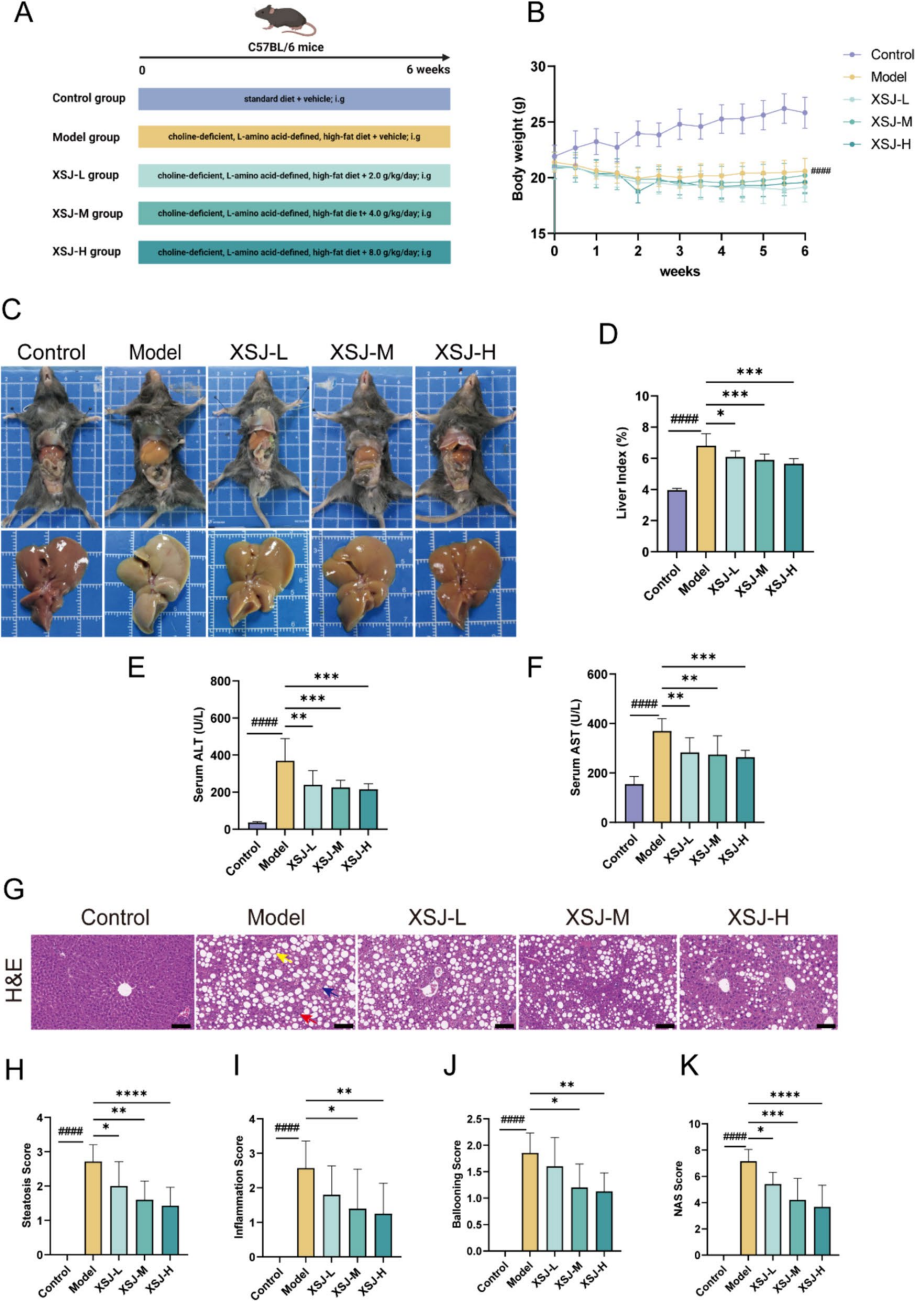
XSJ alleviates liver injury in MASH mice. **A** Schematic diagram and timeline of the mouse experiment. Created in BioRender.com (https://BioRender.com/2y6qd0f). **B** Body weights of the mice during the dietary period (n = 10). **C** Changes in the appearance of the mice and livers. **D** Liver index (n = 10). **E** Serum ALT enzyme activity (n = 10). **F** Serum AST enzyme activity (n = 10). **G** H&E staining of liver tissues (steatosis: yellow arrow; hepatocellular ballooning: blue arrow; inflammatory cell infiltration: red arrow; scale bar: 100 µm). **H** Steatosis score (n = 3). **I** Inflammation score (n = 3). **J** Ballooning score (n = 3). **K** NAS score (n = 3). The data are presented as the mean ± SEM. Compared with the control group, ####*p* < 0.0001; compared with the model group, *p < 0.05, **p < 0.01, ***p < 0.001, ****p < 0.0001

To investigate the therapeutic potential of XSJ in the treatment of MASH, mice were subjected to gavage treatment with XSJ at doses of 2.0, 4.0, and 8.0 g/kg while consuming a CDAHFD for 6 weeks (Fig. 3A). We monitored the changes in the body weight of the mice. Compared with the control diet, the CDAHFD led to a gradual decrease in body weight. However, XSJ administration did not cause significant changes in body weight compared with that observed in the model group (Fig. 3B). The CDAHFD significantly increased the liver index, which was significantly improved by XSJ treatment (Fig. 3D). The livers of control mice appeared dark red, smooth and shiny surface, and sharp edges, whereas the livers of model group mice exhibited typical steatotic characteristics, including enlargement, yellowish discoloration, and greasy texture. XSJ administration partially alleviated these symptoms (Fig. 3C). ALT and AST are markers of liver injury. The results revealed that the CDAHFD significantly elevated ALT and AST activities, which were significantly reduced by XSJ treatment (Fig. 3E-F). Histological analysis further confirmed the protective effects of XSJ on MASH. Liver H&E staining revealed that the CDAHFD induced hepatic steatosis, hepatocellular ballooning, and inflammatory cell infiltration, all of which were partially alleviated by XSJ treatment (Fig. 3G). Additionally, the scores for steatosis, hepatocellular ballooning, inflammation, and the NAFLD activity score (NAS) were significantly reduced, further supporting the protective effects of XSJ on MASH (Fig. 3H-K). In summary, XSJ effectively alleviates liver injury induced by a CDAHFD in mice.

### XSJ improves CDAHFD-induced hepatic lipid accumulation and fibrosis

**Fig. 4:**
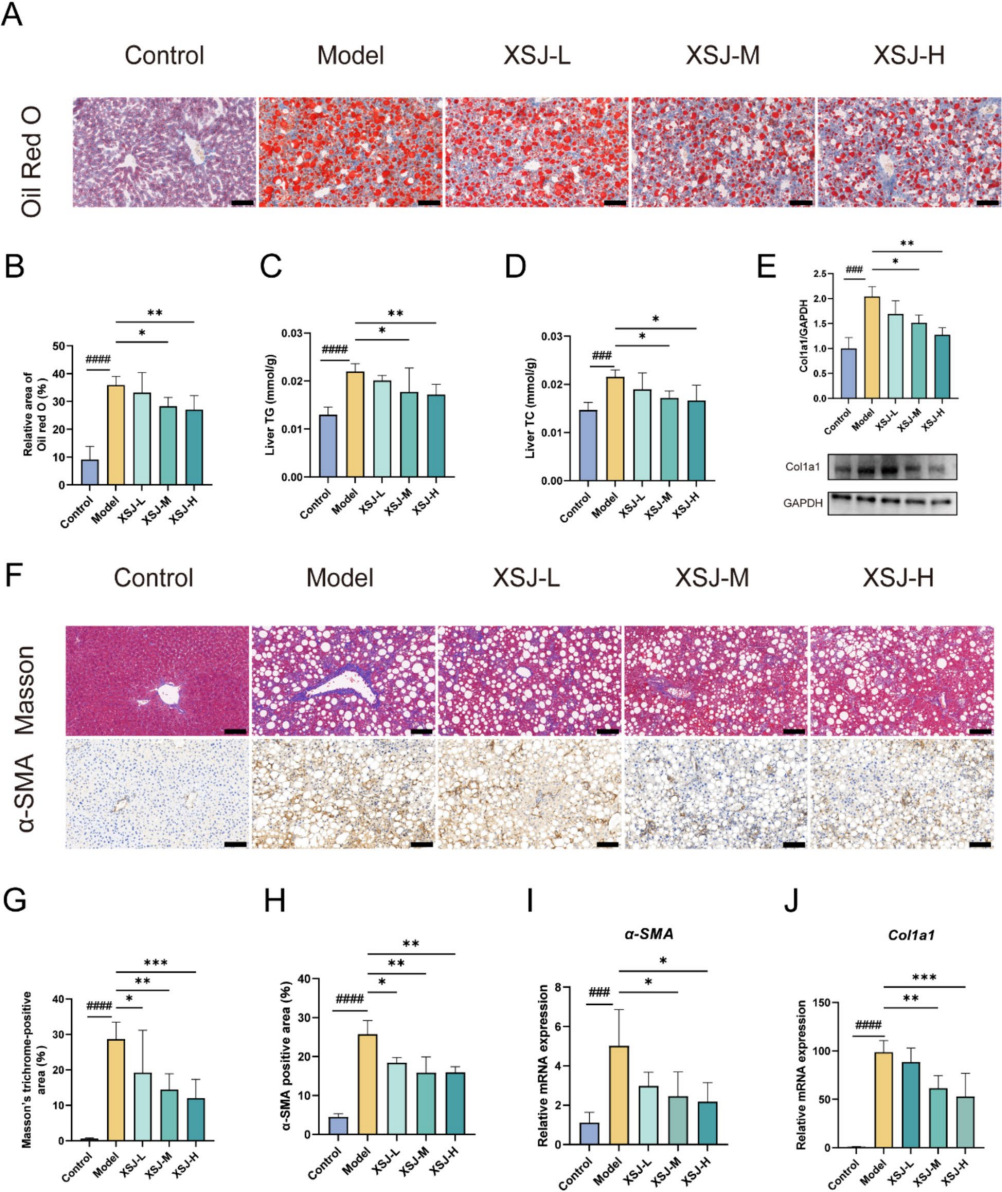
XSJ ameliorates hepatic lipid accumulation and fibrosis in MASH mice. **A** Oil Red O staining of the liver (scale bar: 100 µm). **B** Quantification of the Oil Red O-positive area (n = 3). **C** Liver TG levels (n = 6). **D** Liver TC levels (n = 6). **E** Col1a1 protein expression in the liver was quantified by Western blotting (n = 3). **F** Liver Masson staining and α-SMA immunohistochemical staining (scale bar: 100 µm). **G** Quantification of the Masson-stained area (n = 3). **H** Quantification of the α-SMA-positive area (n = 3). **I** Relative mRNA levels of *α-SMA* in the liver (n = 6). **J** Relative mRNA levels of *Col1a1* in the liver (n = 6). The data are presented as the mean ± SEM. Compared with the control group, ###*p* < 0.001, ####*p* < 0.0001; compared with the model group, *p < 0.05, **p < 0.01, ***p < 0.001

Lipid accumulation is among the core pathological features of MASH. Oil Red O staining revealed that a large number of lipid droplets accumulated in the livers of the model group mice, whereas XSJ treatment significantly reduced lipid accumulation in the livers of MASH mice (Fig. 4A-B). In the model group, the levels of hepatic TG and TC were significantly elevated, whereas XSJ administration prevented the abnormal increase in TG and TC levels (Fig. 4C-D). Western blotting results revealed that XSJ intervention reduced the expression of the Col1a1 protein (Fig. 4E). We subsequently performed Masson staining to assess the degree of liver fibrosis. The CDAHFD significantly promoted the deposition of collagen fibers in the liver, whereas XSJ treatment significantly reduced the excessive deposition of collagen fibers (Fig. 4F-G). Immunohistochemical analysis further demonstrated that XSJ treatment significantly decreased the expression of the activated hepatic stellate cell marker α-SMA (Fig. 4F and H). Consistent with these findings, the mRNA expression of the fibrosis-related genes *α-SMA* and *Col1α1* was significantly upregulated in the livers of MASH mice, while XSJ reversed their overexpression (Fig. 4I-J). In conclusion, XSJ has promising therapeutic potential for alleviating lipid accumulation and liver fibrosis induced by a CDAHFD.

### XSJ reduces CDAHFD-induced inflammation and oxidative stress

**Fig. 5:**
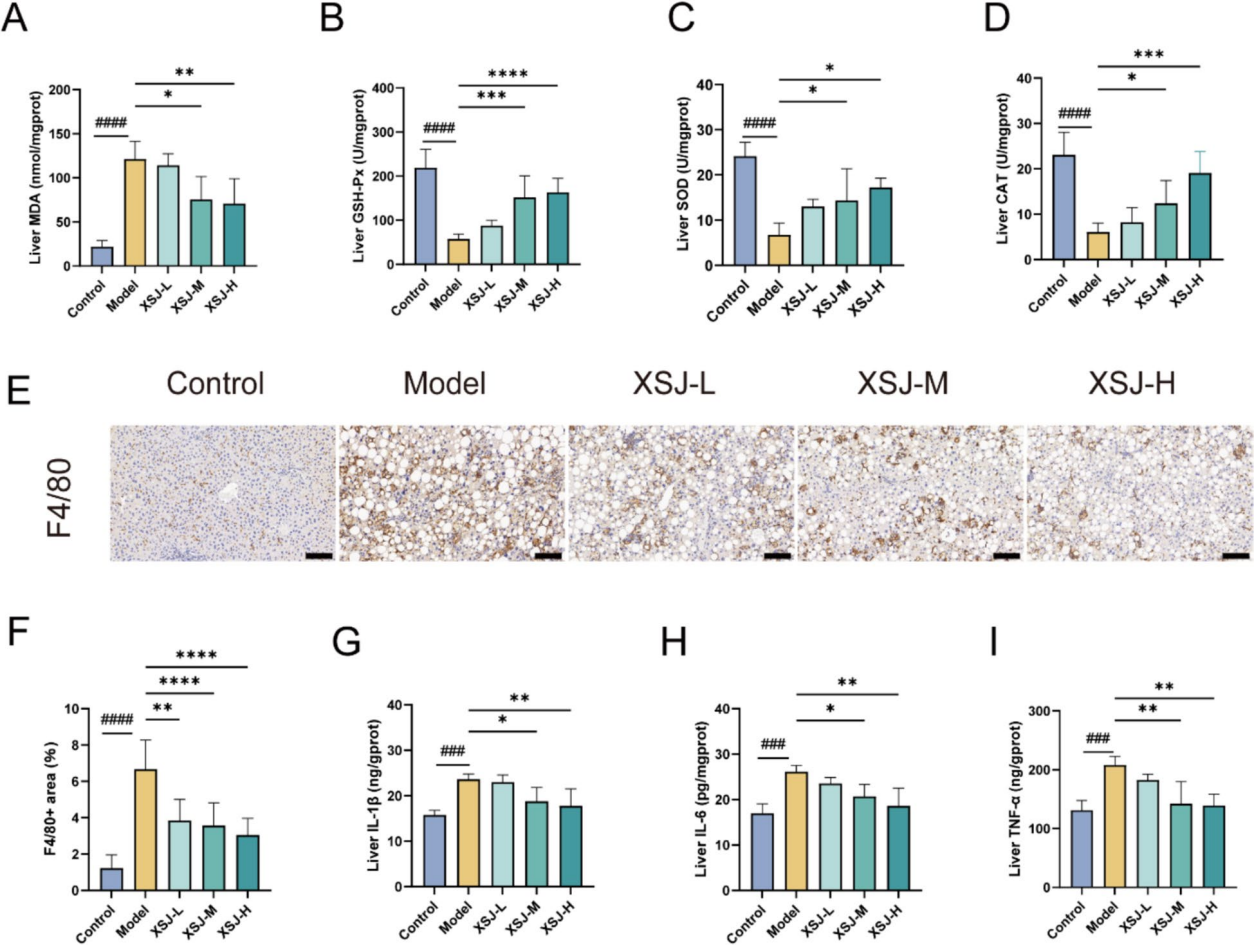
XSJ ameliorates hepatic inflammation and oxidative stress in MASH mice. **A** Liver MDA levels (n = 6). **B** Liver GSH-Px activity (n = 6). **C** Liver SOD activity (n = 6). **D** Liver CAT activity (n = 6). **E** Liver F4/80 immunohistochemical staining (scale bar: 100 µm). **F** Quantification of the F4/80-positive area in the liver (n = 3). **G** Liver IL-1β levels (n = 6). **H** Liver IL-6 levels (n = 6). **I** Liver TNF-α levels (n = 6). The data are presented as the mean ± SEM. Compared with the control group, ###*p* < 0.001, ####*p* < 0.0001; compared with the model group, *p < 0.05, **p < 0.01, ***p < 0.001, ****p < 0.0001

Inflammatory responses and oxidative stress are key pathological mechanisms influencing the progression of MASH. Oxidative stress, caused by an imbalance in the production and clearance of reactive oxygen species, can trigger pathological progression from steatosis to MASH. MDA is a product of lipid peroxidation, reflecting the degree of tissue oxidative damage. Compared with the control diet, the CDAHFD significantly increased the liver MDA content, while XSJ treatment reduced its level (Fig. 5A). We subsequently measured the activities of key antioxidant enzymes in the liver. The CDAHFD significantly decreased the activities of hepatic GSH-Px, SOD, and CAT, whereas XSJ treatment restored these enzyme activities to varying degrees (Fig. 5B-D). To assess the effects of XSJ on liver inflammatory status, we examined the infiltration of F4/80-positive macrophages. The results showed that XSJ significantly reduced macrophage infiltration in the liver induced by the CDAHFD (Fig. 5E-F). Furthermore, we measured the expression levels of inflammatory cytokines. The CDAHFD significantly increased the expression of IL-1β, IL-6, and TNF-α in the liver, whereas XSJ treatment reduced the levels of these inflammatory cytokines (Fig. 5G-I). These results indicate that XSJ exerts protective effects against MASH by inhibiting oxidative stress and inflammatory responses.

### XSJ regulates CDAHFD-induced liver metabolic dysfunction

**Fig. 6:**
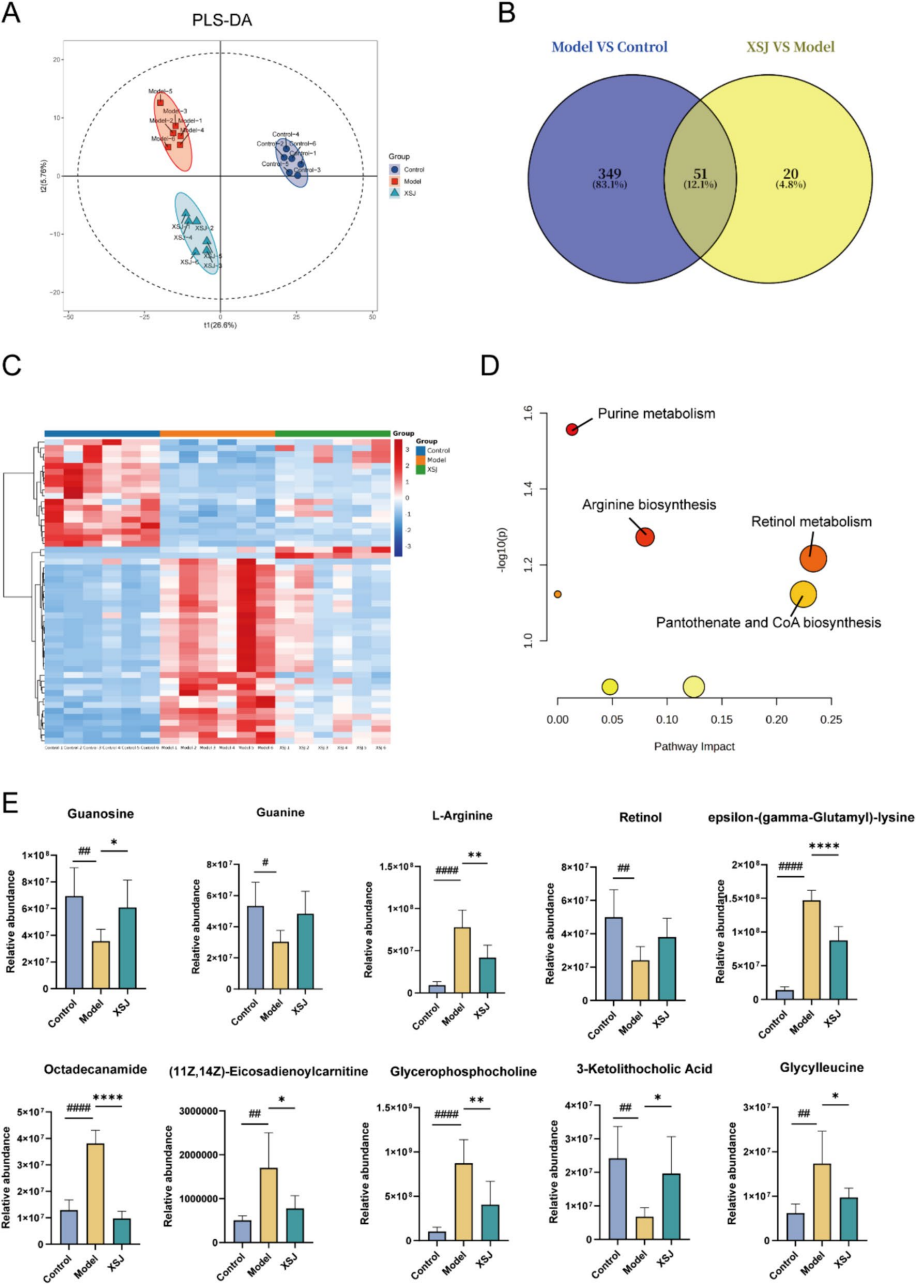
XSJ ameliorates liver metabolic dysfunction. **A** PLS-DA analysis. **B** Venn diagram of DMs. **C** Heatmap of 51 overlapping DMs. **D** Pathway impact plot of 51 overlapping DMs. **E** Ten representative DMs (n = 6). The data are presented as the mean ± SEM. Compared with the control group, #*p* < 0.05, ##*p* < 0.01, ####*p* < 0.0001; compared with the model group, *p < 0.05, **p < 0.01, ****p < 0.0001

Untargeted metabolomics analysis was performed on liver samples to investigate the effect of XSJ on the hepatic metabolic profile of mice. The results of the PLS-DA model revealed distinct separations among the control group, model group, and XSJ group (Fig. 6A). A total of 400 differential metabolites (DMs) were identified between the model group and the control group. After XSJ intervention, 71 DMs were reversed compared with those in the model group. Venn analysis further revealed 51 overlapping DMs between the two sets of DMs (Fig. 6B-C and Supplementary file 2). The metabolic pathways of these 51 overlapping DMs were analyzed using the MetaboAnalyst 6.0 platform (https://www.metaboanalyst.ca/), which included mainly purine metabolism, arginine biosynthesis, retinol metabolism, and pantothenate and CoA biosynthesis (Fig. 6D). Compared with those in the control group, the levels of metabolites such as L-arginine, epsilon-(gamma-glutamyl)-lysine, octadecanamide, (11Z,14Z)-eicosadienoylcarnitine, glycerophosphocholine, and glycylleucine were significantly increased in the model group, whereas the levels of metabolites such as guanosine, guanine, retinol, and 3-ketolithocholic acid were significantly decreased. XSJ administration effectively counteracted these metabolic abnormalities (Fig. 6E), supporting its potential role in regulating liver metabolic dysfunction.

### Transcriptomic analysis of XSJ intervention in MASH

**Fig. 7:**
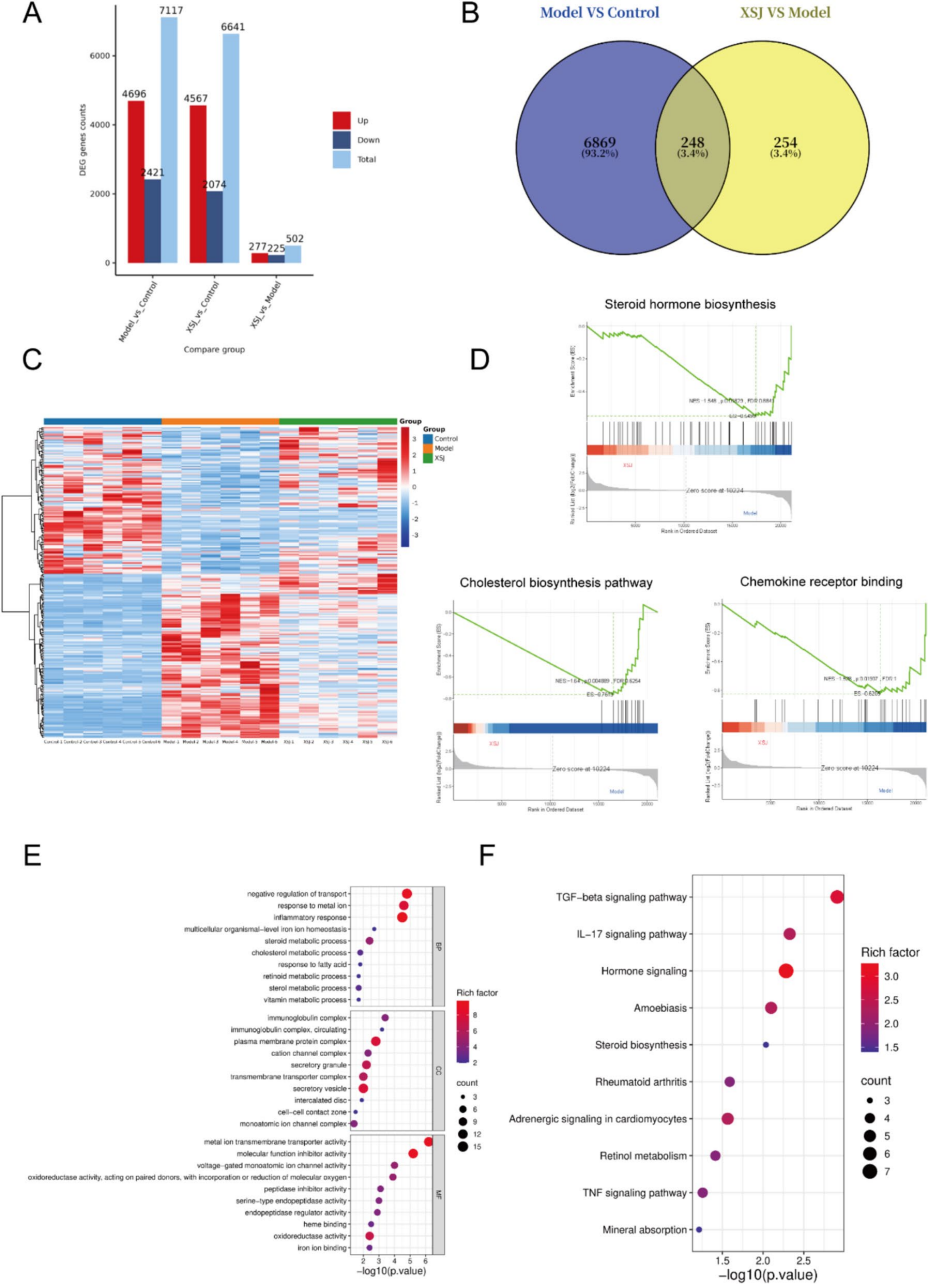
Transcriptomic analysis of the liver. **A** Number of DEGs in different comparison groups. **B** Venn analysis of DEGs. **C** Heatmap of 248 overlapping DEGs. **D** GSEA of the XSJ group vs. the model group. **E** GO analysis of the overlapping DEGs. **F** KEGG pathway enrichment analysis of the overlapping DEGs

To further elucidate the molecular mechanisms by which XSJ ameliorates the MASH phenotype, we conducted a comprehensive transcriptomic analysis of mouse livers. We identified 7,117 DEGs when we compared the model group to the control group (4,696 upregulated and 2,421 downregulated), whereas 502 DEGs were found in the XSJ group compared with the model group (277 upregulated and 225 downregulated) (Fig. 7A). A Venn diagram analysis was subsequently conducted to determine the overlap, revealing 248 common DEGs that were significantly modulated by XSJ treatment (Fig. 7B). To intuitively illustrate the changes in their expression patterns, we constructed a heatmap of these 248 overlapping DEGs, which offered important insights into the molecular action of XSJ in MASH (Fig. 7C and Supplementary file 3). The results of gene set enrichment analysis (GSEA) indicated that XSJ intervention led to the significant downregulation of pathways involved in processes such as steroid hormone biosynthesis, cholesterol biosynthesis, and chemokine receptor binding (Fig. 7D). GO enrichment analysis of the 248 overlapping DEGs revealed significant enrichment in terms including inflammatory response, steroid metabolic process, cholesterol metabolic process, retinoid metabolic process, metal ion transmembrane transporter activity, heme binding, oxidoreductase activity, and iron ion binding (Fig. 7E). Furthermore, KEGG pathway enrichment analysis revealed that the DEGs were predominantly enriched in the TGF-beta signaling pathway, IL-17 signaling pathway, hormone signaling, steroid biosynthesis, retinol metabolism, TNF signaling pathway, and mineral absorption (Fig. 7F). These findings suggest that XSJ may ameliorate the pathological progression of MASH by modulating these key pathways.

### XSJ improves CDAHFD-induced liver fibrosis by inhibiting the activity of the TGF-β1/Smads and PI3K/AKT/Hmox1 signaling pathways

**Fig. 8:**
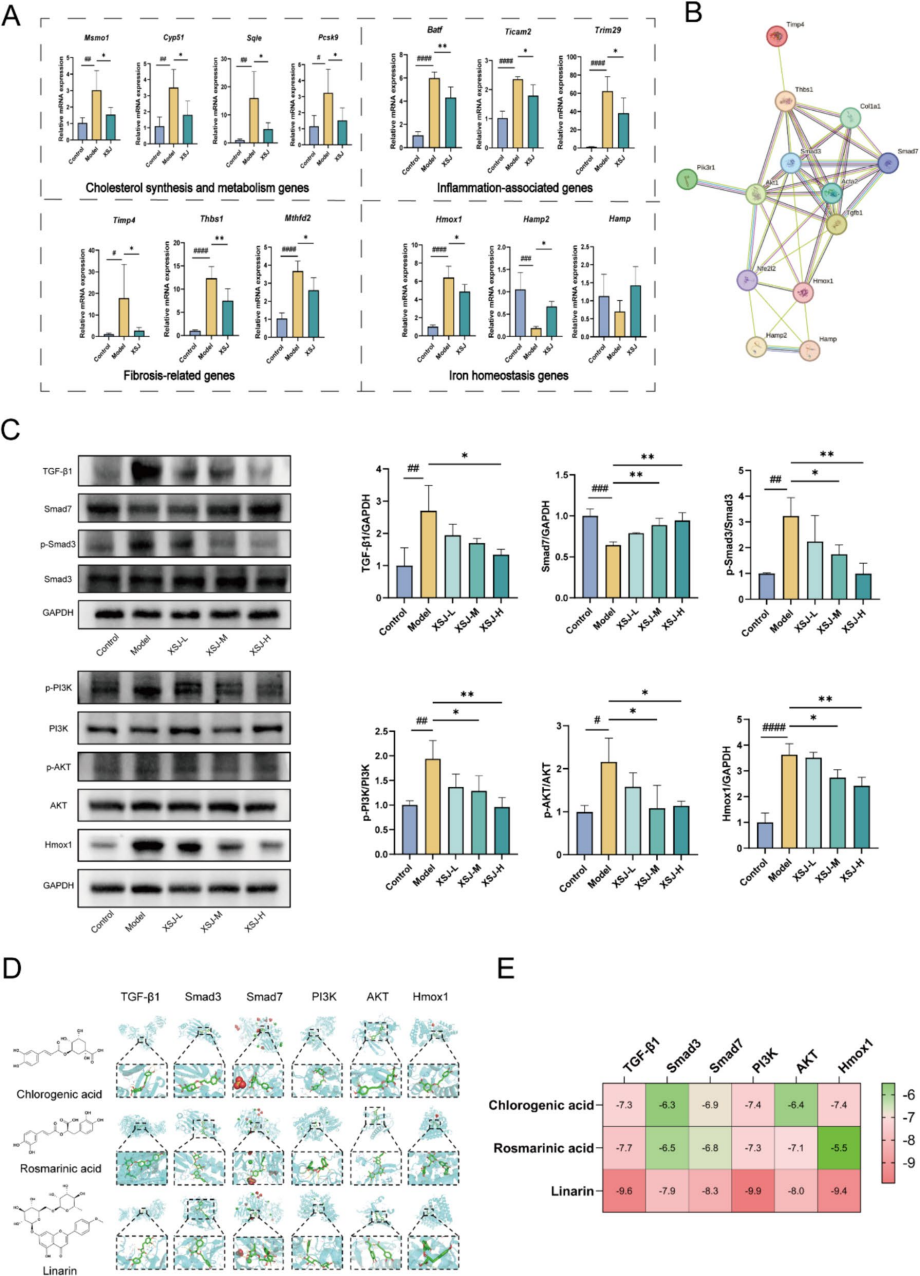
XSJ ameliorates liver fibrosis by inhibiting the TGF-β1/Smads and PI3K/AKT/Hmox1 pathways. **A** RT-qPCR validation of the DEGs (n = 6). **B** PPI network based on the integrated analysis of network pharmacology and transcriptomics. **C** Representative Western blotting and quantification of proteins in the TGF-β1/Smads and PI3K/AKT/Hmox1 pathways (n = 3). **D** Molecular docking images of chlorogenic acid, rosmarinic acid, and linarin with their target proteins. **E** Heatmap of molecular binding energies (kcal/mol). The data are presented as the mean ± SEM. Compared with the control group, #*p* < 0.05, ##*p* < 0.01, ####*p* < 0.0001; compared with the model group, *p < 0.05, **p < 0.01

To validate the results of the transcriptomic analysis, we performed RT-qPCR to verify the selected DEGs. The results showed that XSJ downregulated the expression of cholesterol synthesis-related genes (*Msmo1*, *Sqle*, and *Cyp51*) and metabolism-related genes (*Pcsk9*) induced by the CDAHFD. Moreover, XSJ downregulated the expression of inflammation-related genes (*Batf*, *Ticam2*, and *Trim29*) and fibrosis-related genes (*Timp4*, *Thbs1*, and *Mthfd2*). Notably, XSJ reversed the expression of iron homeostasis-related genes (*Hmox1*, *Hmap2*, and *Hamp*) induced by the CDAHFD (Fig. 8A). Through network pharmacology prediction and liver transcriptomic analysis, we found that the TGF-beta signaling pathway plays a crucial regulatory role in the improvement of CDAHFD-induced MASH by XSJ. In the liver tissue of the model group, the expression of TGF-β1 and phosphorylated Smad3 significantly increased, whereas the expression of Smad7 decreased. After XSJ intervention, the expression levels of these molecules increased, suggesting that XSJ might block the progression of liver fibrosis by inhibiting the TGF-β1/Smads signaling pathway (Fig. 8C). Notably, protein–protein interaction (PPI) network analysis, which combined the results of network pharmacology and transcriptomics, revealed that the PI3K/AKT and Nrf2/Hmox1 pathways might be closely related to liver fibrosis induced by CDAHFD (Fig. 8B). PI3K activates AKT through phosphorylation, and activated AKT promotes the nuclear translocation of the transcription factor Nrf2, thereby promoting the expression of Hmox1 [22]. On this basis, we further verified whether XSJ improved liver fibrosis through the PI3K/AKT/Hmox1 pathway via Western blotting experiments. The results revealed that XSJ significantly reduced the protein expression levels of p-PI3K, p-AKT, and Hmox1 in the liver tissue of the MASH model (Fig. 8C). Chlorogenic acid, rosmarinic acid, and linarin are quality control markers of XSJ, as specified in the Chinese Pharmacopoeia. We selected these compounds and the validated pathways for molecular docking. The docking results demonstrated favorable binding, particularly for linarin, further indicating that XSJ exerts its effects by modulating these pathways (Fig. 8D-E).

### Correlation analysis among biochemical indicators, core targets, and metabolites

**Fig. 9:**
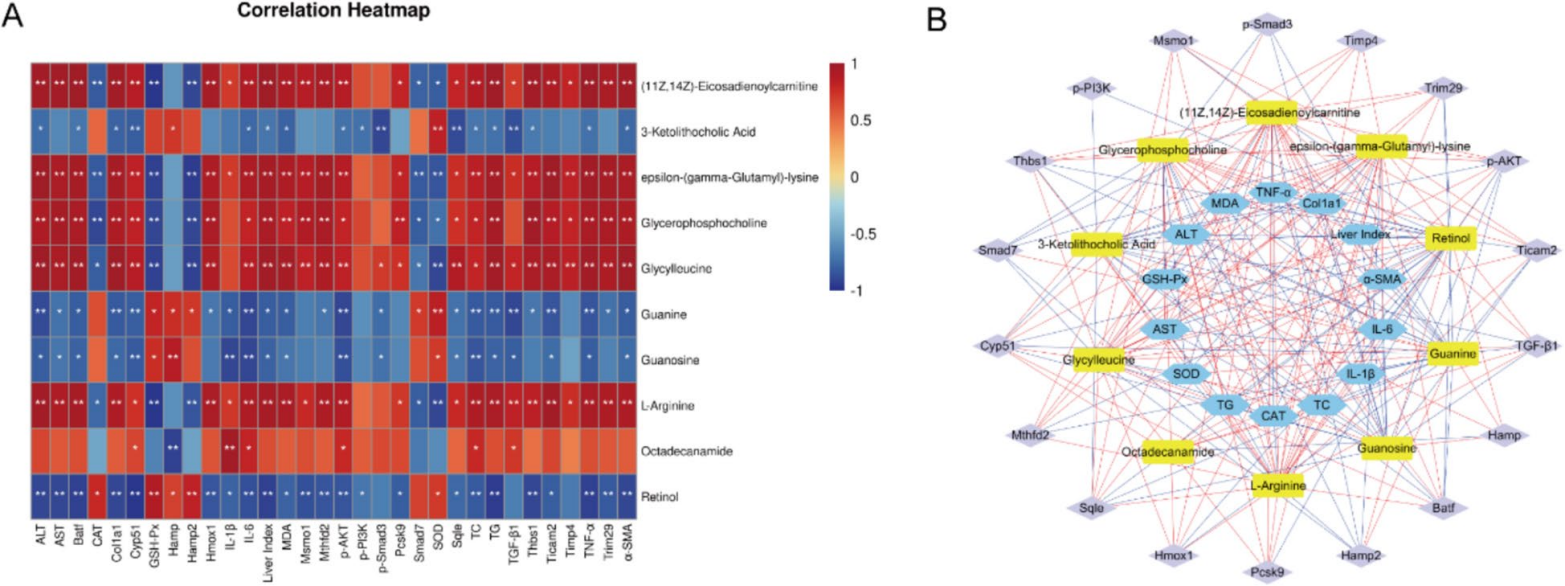
Spearman correlation analysis. **A** Heatmap of correlations between biochemical indicators, core targets, and metabolites. * indicates *p* < 0.05, and ** indicates *p* < 0.01. **B** Network of correlations among biochemical indicators, core targets, and metabolites (only correlations with *p* < 0.05 are shown). Purple nodes represent core targets, blue nodes represent biochemical indicators, and yellow nodes represent metabolites. Red lines indicate positive correlations, and blue lines indicate negative correlations

To further elucidate the mechanism by which XSJ alleviates MASH, a comprehensive Spearman correlation analysis was conducted on MASH-related biochemical indicators, core targets, and DMs. The intensity of the correlation is represented by color shading, with red indicating a positive correlation and blue indicating a negative correlation. As illustrated in Fig. 9A, metabolites such as L-arginine, epsilon-(gamma-glutamyl)-lysine, octadecanamide, (11Z,14Z)-eicosadienoylcarnitine, and glycerophosphocholine were significantly positively correlated with multiple biochemical markers and targets. For instance, epsilon-(gamma-glutamyl)-lysine demonstrated a strong positive correlation with fibrosis markers such as Col1a1 and α-SMA. Conversely, metabolites such as guanosine, guanine, retinol, and 3-ketolithocholic acid were significantly negatively correlated with various biochemical markers and targets. For example, retinol was negatively correlated with TG and α-SMA levels. To visually illustrate these correlations, we constructed a comprehensive network integrating MASH-related biochemical markers, core targets, and liver metabolomic differences (Fig. 9B).

Overall, these correlations not only reveal the potential roles of various metabolites and targets in the pathogenesis of MASH but also provide important insights for future research. In particular, metabolites that are strongly correlated with multiple indicators may represent key therapeutic targets for future interventions and warrant further investigation. These findings enhance our understanding of how XSJ alleviates MASH symptoms by modulating these key metabolites and provide a scientific basis for the development of novel therapeutic strategies.

## Discussion

**Fig. 10:**
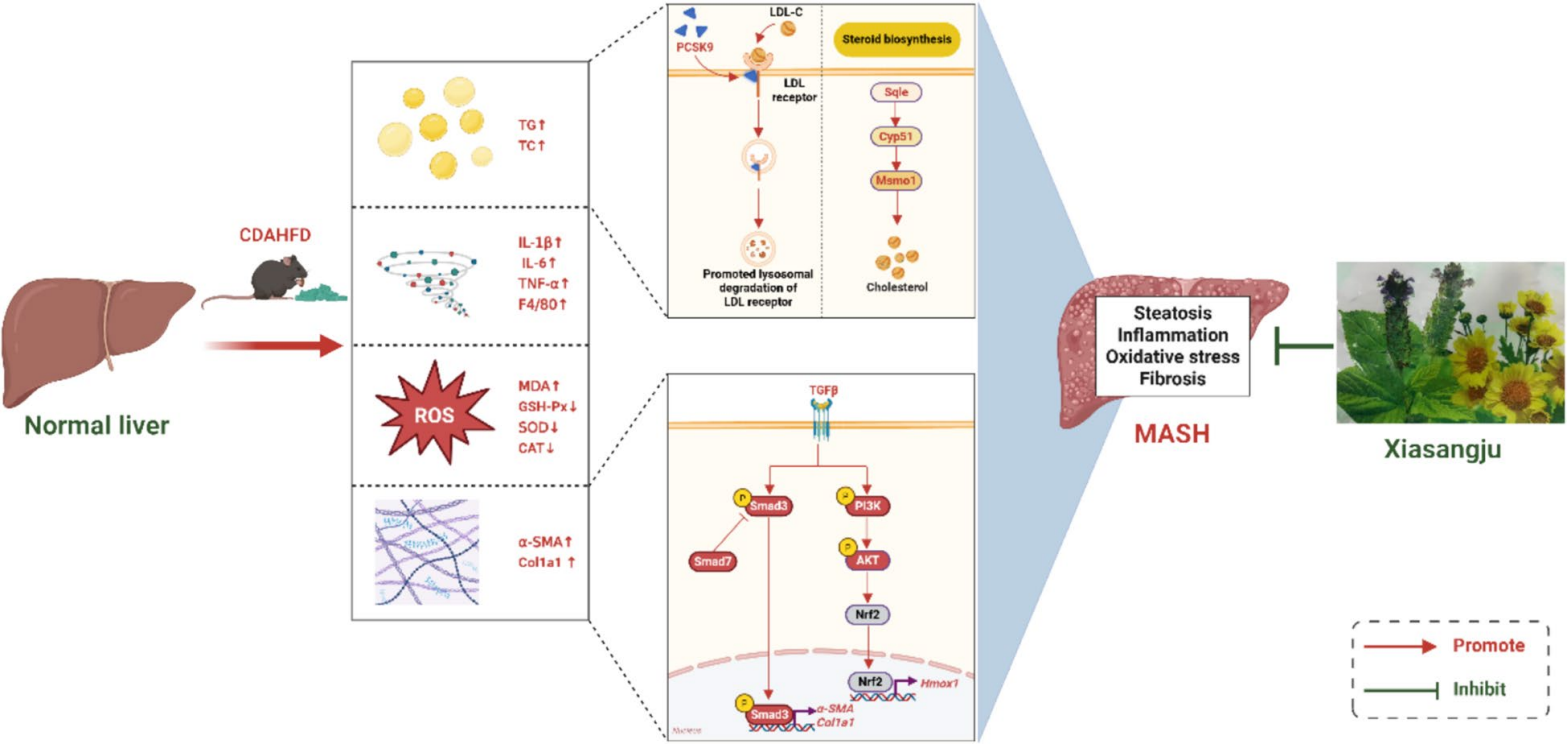
Schematic diagram of the potential mechanism of XSJ in improving MASH. XSJ can ameliorate lipid accumulation, the inflammatory response, oxidative stress, and liver fibrosis in CDAHFD-induced MASH through a variety of mechanisms. On the one hand, XSJ alleviates steatosis by inhibiting the cholesterol biosynthesis and metabolism pathways. On the other hand, XSJ can inhibit the TGF-β1/Smads signaling pathway and the PI3K/AKT/Hmox1 pathway, thereby suppressing the progression of liver fibrosis. Created in BioRender (https://BioRender.com/w172qbf)

MASH is a complex liver disease driven by multiple pathological processes, including lipid accumulation, oxidative stress, inflammation, and fibrosis. This study provides the first demonstration of multidimensional improvements by the TCM formula XSJ in CDAHFD-induced MASH, offering strong experimental support for its potential as a therapeutic drug (Fig. 10).

TCM formulas are characterized by their complex composition and multitarget, multipathway mechanisms, demonstrating considerable potential in the treatment of MASH. Through UPLC-Q-TOF–MS analysis, we identified 74 chemical components in XSJ. Through network pharmacology analysis, we subsequently identified potential target genes associated with the ability of XSJ to ameliorate MASH. These targets are involved primarily in biological processes closely related to liver fibrosis, such as the PI3K-AKT and TGF-β signaling pathways and ECM-receptor interactions. These findings suggest that XSJ may exert its potential therapeutic effects on MASH by inhibiting the activation of fibrosis-related signaling pathways, laying a theoretical foundation for subsequent in-depth research into its pharmacological mechanisms.

Untargeted metabolomics analysis provides a crucial metabolic context for the antifibrotic effects of XSJ. XSJ reversed a variety of metabolic abnormalities induced by a CDAHFD, particularly by reducing the levels of L-arginine and epsilon-(gamma-glutamyl)-lysine, both of which are closely associated with fibrosis. L-arginine is a key regulator of TGF-β-induced fibroblast activation [23], while epsilon-(gamma-glutamyl)-lysine, a transglutaminase-catalyzed cross-linking product, stabilizes the extracellular matrix [24, 25]. These findings indicate that XSJ not only inhibits pro-fibrotic signaling but also removes the “fuel” for fibrosis at the level of metabolic substrates. Furthermore, XSJ increased retinol levels, and retinol may contribute to the antifibrotic effect by regulating lipid metabolism and maintaining the quiescent state of HSCs through its active metabolite retinoic acid [26].

RT-qPCR analysis confirmed that XSJ inhibited the expression of fibrosis-associated genes (*Timp4*, *Thbs1*, and *Mthfd2*). Tissue inhibitor of metalloproteinases 4 (Timp4) modulates ECM turnover, and its absence improves obesity and related complications [27]. Thrombospondin 1 (Thbs1) is pivotal for TGF-β1 activation, and interrupting their interaction is a potential antifibrotic strategy [28]. By inducing methylenetetrahydrofolate dehydrogenase 2 (Mthfd2), TGF-β enhances mitochondrial one-carbon metabolism, which is essential for glycine and collagen synthesis [29]. Pharmacological inhibition of Mthfd2 can improve the fibrotic response [30]. Additionally, iron homeostasis is linked to liver fibrosis [31], and XSJ reversed the expression of related genes (*Hmox1*, *Hamp*, *Hamp2*). Hepcidin (Hamp), a key iron metabolism regulator, acts as an endogenous antifibrotic factor in HSC activation [32], and its overexpression alleviates steatohepatitis and fibrosis [33]. Notably, XSJ reduced CDAHFD-induced Hmox1 expression. While Hmox1 is generally protective via antioxidant effects [34], its excessive upregulation can cause iron overload, trigger ferroptosis, and worsen fibrosis [35, 36]. Transcriptomic and network pharmacology analyses revealed the TGF-β1/Smads pathway as a crucial pathway affected by XSJ. Furthermore, PPI network analysis revealed that the PI3K/AKT and Nrf2/Hmox1 pathways also play significant roles in the improvement of MASH by XSJ. The PI3K/AKT pathway is critical for alleviating liver fibrosis [37], and its inhibition improves CDAHFD-induced fibrosis [38, 39]. Moreover, activation of the PI3K/AKT pathway can promote the nuclear translocation and transcriptional activity of Nrf2, thereby activating the overexpression of Hmox1 [40]. Hmox1 then releases free iron via heme catabolism, promoting ferroptosis and fibrosis progression [41]. Our results indicate that XSJ also protects against MASH by suppressing the PI3K/AKT/Hmox1 axis. In summary, XSJ may exert antifibrotic effects by inhibiting the TGF-β1/Smads and PI3K/AKT/Hmox1 pathways. Critically, these two pathways do not operate independently; TGF-β1 can activate the PI3K/AKT/Hmox1 pathway in a Smad-independent manner. The simultaneous inhibition of these two pathways by XSJ effectively blocks the further progression of fibrosis.

XSJ also ameliorated hepatic steroid hormone and cholesterol biosynthesis. This finding was confirmed by RT-qPCR, which revealed that XSJ downregulated the expression of cholesterol synthesis genes [42] (e.g., *Msmo1*, *Cyp51*, and *Sqle*) and the metabolism-related gene proprotein convertase subtilisin/kexin type 9 (*Pcsk9*) [43], leading to improved lipid metabolism and reduced hepatic steatosis. In addition, KEGG enrichment of the transcriptome highlighted the enrichment of genes related to IL-17 and TNF-α signaling, GO analysis flagged inflammatory-response processes, and GSEA showed that XSJ suppressed the chemokine-binding pathway, collectively revealing broad anti-inflammatory activity at the molecular level. RT-qPCR further corroborated the transcriptomic data: XSJ decreased the expression of the inflammatory genes *Batf*, *Ticam2* and *Trim29*. Basic leucine zipper ATF-like transcription factor (Batf) promotes inflammation by driving Th17 differentiation [44]; Toll-interleukin 1 receptor domain containing adaptor protein 2 (Ticam2) contributes to inflammatory disorders via TLR signaling [45]; and Tripartite motif-containing 29 (Trim29) facilitates pyroptosis through NF-κB/NLRP3 activation [46]. Moreover, XSJ elevated 3-ketolithocholic acid, a bile acid with anti-inflammatory, immunomodulatory and metabolic potential that curbs Th17 differentiation and attenuates inflammation [47], providing metabolic support for the anti-inflammatory effects of XSJ.

XSJ is composed of *Prunella vulgaris* L., *Morus alba* L., and *Chrysanthemum indicum* L., and its beneficial effect on MASH most likely arises from the synergistic action of multiple bioactive compounds. This study demonstrates the multitarget, multipathway synergistic mode of action of XSJ. Flavonoid components, such as linarin and rutin, which have potent antioxidant and anti-inflammatory properties, may inhibit upstream inflammatory responses and oxidative damage by scavenging free radicals and alleviating oxidative stress [48, 49]. Phenolic acid components, such as chlorogenic acid and rosmarinic acid, may play a core regulatory role by directly inhibiting TGF-β-induced HSC activation and fibrotic signaling pathways [50, 51]. Triterpenoid components, such as ursolic acid, may in turn improve the metabolic foundation of MASH by regulating lipid metabolism [52]. This multipathway approach gives XSJ an advantage over single-target drugs in addressing the complex pathology of MASH. As a widely used and safe medicinal food homology formula, XSJ represents a promising candidate for the treatment of MASH. Nevertheless, the current work is limited primarily to pharmacodynamic evaluation and preliminary exploration of mechanisms. Future research should focus on analyzing the active ingredients and their relative contributions, deeply exploring the synergistic mechanism among key components, and further strengthening the correlation between the active ingredients of XSJ and its pharmacological mechanism. Moreover, designing rigorous and standardized trials to systematically evaluate the clinical efficacy of XSJ in MASH is essential.

## Conclusion

In summary, this study systematically investigated the therapeutic effects of the TCM compound XSJ on MASH using a multiomics approach. UPLC-Q-TOF-MS analysis revealed 74 chemical constituents in XSJ, with phenolic acids and flavonoids being the major active components. Network pharmacology analysis predicted key targets of XSJ in the treatment of MASH and revealed its potential therapeutic effects through the regulation of the PI3K-AKT and TGF-β signaling pathways. In a CDAHFD-induced MASH mouse model, XSJ ameliorated liver injury, lipid accumulation, inflammatory responses, oxidative stress, and liver fibrosis. Untargeted metabolomics analysis revealed that XSJ could modulate multiple metabolic pathways, including purine metabolism, arginine biosynthesis, retinol metabolism, and pantothenate and CoA biosynthesis, thereby ameliorating hepatic metabolic disturbances. Transcriptomic analysis further revealed the regulatory effects of XSJ on gene expression in the liver, particularly its significant impact on genes involved in cholesterol biosynthesis, inflammatory responses, and fibrosis. Moreover, XSJ attenuated the progression of liver fibrosis by inhibiting the TGF-β1/Smads and PI3K/AKT/Hmox1 signaling pathways. The findings of this study provide scientific evidence supporting the potential of XSJ as a therapeutic agent for MASH, revealing its multicomponent, multitarget, and multipathway mechanisms. These insights offer valuable guidance for the development of novel therapeutic strategies.

## Supplementary Information

Supplementary file 1.

Supplementary file 2.

Supplementary file 3.

## Data Availability

No datasets were generated or analysed during the current study.

## Abbreviations

XSJ: Xiasangju
MASH: Metabolic dysfunction associated steatohepatitis
MASLD: Metabolic dysfunction-associated steatotic liver disease
CDAHFD: Choline-deficient, L-amino acid-defined, high-fat diet
UPLC-Q-TOF–MS: Ultrahigh performance liquid chromatography with quadrupole time-of-flight mass spectrometry
TCM: Traditional Chinese medicine
ECM: Extracellular matrix
HSC: Hepatic sellate cell
α-SMA: Alpha smooth muscle actin
Col1a1: Collagen type I alpha 1
TGF-β1: Transforming growth factor beta 1
Smad3: Mothers against decapentaplegic homolog 3
PI3K: Phosphoinositide-3 kinase
AKT: Protein kinase B
Hmox1: Heme oxygenase 1
Nrf2: Nuclear factor erythroid 2-related factor 2
ALT: Alanine aminotransferase
AST: Aspartate aminotransferase
TC: Total cholesterol
TG: Triglycerides
MDA: Malondialdehyde
SOD: Superoxide dismutase
CAT: Catalase
GSH-Px: Glutathione peroxidase
IL-1β: Interleukin-1β
IL-6: Interleukin-6
TNF-α: Tumor necrosis factor-α
GO: Gene ontology
KEGG: Kyoto encyclopedia of genes and genomes
RT-qPCR: Real-time quantitative polymerase chain reaction
PLS-DA: Partial least squares discriminant analysis
DMs: Differential metabolites
DEGs: Differentially expressed genes
Msmo1: Methylsterol monooxygenase 1
Cyp51: Cytochrome P450 51
Sqle: Squalene monooxygenase
PCSK9: Proprotein convertase subtilisin/kexin type 9
Batf: Basic leucine zipper ATF-Like transcription factor
Ticam2: Toll-interleukin 1 receptor domain containing adaptor protein 2
Trim29: Tripartite motif-containing 29
Timp4: Tissue inhibitor of metalloproteinases 4
Thbs1: Thrombospondin 1
Mthfd2: Methylenetetrahydrofolate dehydrogenase 2
PPI: Protein–protein interaction
