# Supplementary material for: Integrative multiomics analysis reveals the ameliorative effects of Xiasangju on metabolic dysfunction-associated steatohepatitis
Source: Chin Med. 2026 Jan 4;21:3. doi: 10.1186/s13020-025-01275-y (PMC12765294; doi:10.1186/s13020-025-01275-y)
Supplement: Supplementary file 1 [file 13020_2025_1275_MOESM1_ESM.docx]

**Chemical Components of XSJ**

| **NO.** | **t_R_/min** | **Formula** | **Predicted**  **(m/z)** | **Measured**  **(m/z)** | **Error(ppm)** | **MS/MS** | **Identiﬁcation** |
| --- | --- | --- | --- | --- | --- | --- | --- |
| 1 | 0.75 | C_4_H_6_O_5_ | 133.0142 | 133.0142 | 0.00 | 115.0082,89.0314,73.0018,71.0225 | Malic acid |
| 2 | 1.38 | C_7_H_6_O_5_ | 169.0142 | 169.0152 | 5.92 | 125.0291,124.0206,107.0200,79.0272,69.0437 | Gallic acid* |
| 3 | 2.21 | C_8_H_8_O_4_ | 167.035 | 167.0350 | 0 | 123.0487,108.0274,93.0397 | Vanillic acid |
| 4 | 2.31 | C_9_H_10_O_5_ | 197.0455 | 197.0460 | 2.54 | 179.0326,151.0410,135.0474,123.0490,73.0015 | Danshensu |
| 5 | 2.47 | C_7_H_6_O_4_ | 153.0193 | 153.0196 | 1.96 | 109.0347,108.0274,91.0263 | Protocatechuic acid |
| 6 | 3.00 | C_16_H_18_O_9_ | 353.0878 | 353.0877 | -0.28 | 191.0523,179.0332,161.0252,135.0482,93.0424 | Neochlorogenic acid* |
| 7 | 3.21 | C_9_H_10_O_4_ | 181.0506 | 181.0511 | 2.76 | 163.0395,137.0265,135.0474 | Dihydrocaffeic acid |
| 8 | 3.47 | C_15_H_16_O_9_ | 339.0722 | 339.0712 | -2.95 | 177.0176,133.0324 | Esculin |
| 9 | 3.85 | C_9_H_8_O_3_ | 163.0401 | 163.0404 | 1.84 | 163.0428,119.0542,117.0418,93.0422 | p-Coumaric acid |
| 10 | 3.99 | C_26_H_32_O_14_ | 567.1719 | 567.1704 | -2.64 | 405.0803,243.0559,225.0460 | Mulberroside A |
| 11 | 4.06 | C_7_H_12_O_6_ | 191.0561 | 191.0568 | 3.66 | 173.0430,127.0442,93.0418,85.0372 | Quinic acid |
| 12 | 4.07 | C_16_H_18_O_9_ | 353.0878 | 353.0883 | 1.42 | 191.0518,179.0325,161.0246,135.0484,85.0375 | Chlorogenic acid* |
| 13 | 4.22 | C_10_H_10_O_4_ | 193.0506 | 193.0505 | -0.52 | 149.0614,134.0377 | Ferulic Acid |
| 14 | 4.27 | C_9_H_6_O_4_ | 177.0193 | 177.0199 | 3.39 | 149.0262,133.0327,121.0341,105.0409,89.0476 | Esculetin |
| 15 | 4.29 | C_8_H_8_O_3_ | 151.0401 | 151.0402 | 0.66 | 108.0265,136.0191,91.0259 | Vanillin |
| 16 | 4.36 | C_16_H_18_O_9_ | 353.0878 | 353.0883 | 1.42 | 191.0525,179.0331,135.0482,93.0423 | Cryptochlorogenic acid |
| 17 | 4.40 | C_9_H_8_O_4_ | 179.0350 | 179.0357 | 3.91 | 135.0485,134.0407,71.0199 | Caffeic acid* |
| 18 | 5.09 | C_26_H_30_O_14_ | 565.1563 | 565.1564 | 0.18 | 521.0381,403.0678,241.0396 | Mulberroside F |
| 19 | 5.16 | C_27_H_30_O_16_ | 609.1461 | 609.1467 | 0.98 | 489.0535,447.0502,327.0285,285.0237 | Kaempferol 3,7-di-O-glucoside |
| 20 | 5.43 | C_25_H_24_O_12_ | 515.1195 | 515.1187 | -1.55 | 353.0588,335.0518,191.0526,179.0332,161.0248,135.0482 | Isochlorogenic acid |
| 21 | 5.48 | C_27_H_30_O_15_ | 593.1512 | 593.1496 | -2.70 | 503.0644,473.0591,383.0444,353.0373,325.0482 | Vicenin 2 |
| 22 | 5.86 | C_27_H_30_O_16_ | 609.1461 | 609.1465 | 0.66 | 447.0467,285.0213 | Luteolin-3',7 -di-O-glucoside |
| 23 | 5.93 | C_26_H_28_O_14_ | 563.1406 | 563.1403 | -0.53 | 503.0615,473.0592,443.0535,383,0429,353.0368,297.0547 | Vicenin 3 |
| 24 | 5.92 | C_27_H_30_O_17_ | 625.1410 | 625.1411 | 0.16 | 493.0612,463.0423,301.0142,300.0068,271.0086,255.0167, | Quercetin-3-O-β-gentiobioside |
| 25 | 6.48 | C_9_H_6_O_3_ | 161.0244 | 161.0250 | 3.73 | 133.0306,132.0231,105.0414,77.0493,65.0129 | Umbelliferone* |
| 26 | 6.53 | C_27_H_30_O_16_ | 609.1461 | 609.1475 | 2.30 | 301.0138,300.0075,271.0101,255.0167 | Rutin* |
| 27 | 6.56 | C_26_H_28_O_14_ | 563.1406 | 563.1408 | 0.36 | 473.0603,443.0510,383,0421,353.0375,297.0593 | Schaftoside |
| 28 | 6.58 | C_22_H_18_O_12_ | 473.0725 | 473.0733 | 1.69 | 293.0137,179.0334,149.0111,135.0486 | Chicoric acid |
| 29 | 6.62 | C_27_H_30_O_16_ | 609.1461 | 609.1473 | 1.97 | 447.0467,285.0231,284.0145,255.0163,227.0245 | Luteolin-7,3'-di-O-glucoside |
| 30 | 6.93 | C_27_H_30_O_16_ | 609.1461 | 609.1471 | 1.64 | 301.0120,300.0070,271.0094,255.0169 | Rhodiosin |
| 31 | 6.98 | C_21_H_20_O_12_ | 463.0882 | 463.0886 | 0.86 | 301.0156,300.0078,271.0095,255.0167,151.0048 | Isoquercitrin* |
| 32 | 7.10 | C_21_H_22_O_11_ | 449.1089 | 449.1100 | 2.45 | 287.0394,151.0062,135.0490,107.0204 | Eriodictyol-7-O-glucoside |
| 33 | 7.13 | C_27_H_22_O_12_ | 537.1038 | 537.1052 | 2.61 | 493.0619,339.0234,295.0417,229.0053,159.0449,135.0474,109.0348 | Lithospermic acid |
| 34 | 7.15 | C_21_H_20_O_12_ | 463.0882 | 463.0883 | 0.22 | 301.0155,300.0073,271.0094,255.0173,151.0043 | Hyperoside* |
| 35 | 7.16 | C_27_H_30_O_15_ | 593.1512 | 593.1507 | -0.84 | 285.0239,284.0163,255.0172 | Nicotiflorin |
| 36 | 7.29 | C_21_H_18_O_12_ | 461.0725 | 461.0730 | 1.08 | 285.0224 | Luteolin-7-O-glucuronide |
| 37 | 7.30 | C_21_H_20_O_11_ | 447.0933 | 447.0938 | 1.12 | 285.0218,284.0142,256.0252,151.0043 | Cynaroside* |
| 38 | 7.40 | C_27_H_30_O_15_ | 593.1512 | 593.1503 | -1.52 | 285.0252,284.0145,255.0166 | Luteolin-7-O-rutinoside |
| 39 | 7.57 | C_24_H_26_O_13_ | 521.1301 | 521.1308 | 1.34 | 359.0675,197.0404,179.0322,161.0240,135.0474,123.0490 | Salviaflaside |
| 40 | 7.62 | C_15_H_18_O_9_ | 341.0878 | 341.0878 | 0.00 | 179.0329,135.0478 | Caffeic acid-4-O-glucoside |
| 41 | 7.74 | C_36_H_30_O_16_ | 717.1461 | 717.1475 | 1.95 | 537.0458,519.0391,475.0555,339.0259,243.0193,197.0412 | rabdosiin |
| 42 | 7.83 | C_7_H_6_O_3_ | 137.0244 | 137.0241 | -2.19 | 93.0412,65.0490 | Salicylic acid |
| 43 | 7.89 | C_16_H_18_O_9_ | 353.0878 | 353.0877 | -0.28 | 191.0504,179.0305,135.0465,134.0388 | 1-Caffeoylquinic acid |
| 44 | 7.98 | C_21_H_20_O_11_ | 447.0933 | 447.0931 | -0.45 | 285.0226,284,0147,255.0160,227.0257 | Kaempferol 3-O-glucoside |
| 45 | 8.22 | C_21_H_20_O_11_ | 447.0933 | 447.0919 | -3.13 | 285.0237,284.0156,151.0046 | Kaempferol 7-O-glucoside |
| 46 | 8.23 | C_22_H_22_O_12_ | 477.1038 | 477.1035 | -0.63 | 315.0297,314.0211,285.0232,271.0095,243.0185 | Isorhamnetin 3-O-glucoside |
| 47 | 8.25 | C_21_H_22_O_10_ | 433.1140 | 433.1131 | -2.08 | 271.0466,151.0030 | Prunin |
| 48 | 8.32 | C_21_H_20_O_10_ | 431.0984 | 431.0969 | -3.48 | 269.0308,268.0223,240.0315,151.0048 | Apigenin 7-O-glucoside* |
| 49 | 8.33 | C_21_H_20_O_10_ | 431.0984 | 431.0977 | -1.62 | 269.0311,268.0243,239.0241 | Genistin |
| 50 | 8.42 | C_21_H_18_O_11_ | 445.0776 | 445.0787 | 2.47 | 269.0311,113.0294 | Apigenin-7-O-glucuronide |
| 51 | 8.62 | C_25_H_24_O_12_ | 515.1195 | 515.1190 | -0.97 | 353.0582,191.0516,179.0318,173.0430,135.0472 | Dicaffeoylquinic acid |
| 52 | 8.65 | C_18_H_16_O_8_ | 359.0772 | 359.0767 | -1.39 | 197.0394,179.0312,161.0234,135.0468,133.0313,123.0485,73.0008 | Rosmarinic acid* |
| 53 | 8.78 | C_22_H_20_O_12_ | 475.0882 | 475.0878 | -0.84 | 299.0362,284.0142,161.0231,113,0284 | Diosmetin 7-O- glucuronide |
| 54 | 9.01 | C_26_H_22_O_10_ | 493.1140 | 493.1148 | 1.62 | 313.0486,295.0421,197.0386,185.0208,135.0478,109.0344 | Salvianolic acid A |
| 55 | 9.52 | C_34_H_42_O_19_ | 753.2248 | 753.2291 | 5.71 | 283.0443,268.228 | Acacetin-7-O-(6″-O-α-L-rhamno-pyranosyl)  β-sophoroside |
| 56 | 9.79 | C_21_H_18_O_11_ | 445.0776 | 445.0787 | 2.47 | 269.0303,113.0300 | Baicalin |
| 57 | 9.92 | C_15_H_12_O_6_ | 287.0561 | 287.0565 | 1.39 | 151.0035,135.0475,134.0417,107.0172 | Eriodictyol |
| 58 | 9.93 | C_28_H_32_O_15_ | 607.1668 | 607.1658 | -1.65 | 299.0375,284.0144,269.0672 | Diosmetin-7-O-rutinoside |
| 59 | 10.14 | C_19_H_18_O_8_ | 373.0929 | 373.0927 | -0.54 | 197.0406,179.0325,175.0382,160.0157,135.0472,123.0481,73.0006 | Methyl rosmarinate |
| 60 | 10.19 | C_15_H_10_O_7_ | 301.0354 | 301.0349 | -1.66 | 273.0226,255.0167,178.9952,151.0036,121.0330,107.0190 | Quercetin |
| 61 | 10.23 | C_15_H_10_O_6_ | 285.0405 | 285.0412 | 2.46 | 175.0378,151.0041,133.0321,107.0190 | Luteolin |
| 62 | 10.51 | C_28_H_32_O_14_ | 591.1719 | 591.1721 | 0.34 | 283.0442,268.0236 | Linarin* |
| 63 | 10.62 | C_11_H_12_O_4_ | 207.0663 | 207.0656 | -3.38 | 179.0278,161.0228,135.0462,134.0385,133.0314 | Ethyl caffeate |
| 64 | 11.01 | C_22_H_20_O_11_ | 459.0933 | 459.0930 | -0.65 | 327.1924,283.0431,268.0224 | Acacetin-7-glucuronide |
| 65 | 11.22 | C_15_H_10_O_5_ | 269.0455 | 269.0455 | 0 | 151.0038,117.0386,107.0179 | Apigenin |
| 66 | 11.44 | C_16_H_12_O_6_ | 299.0561 | 299.0559 | -0.67 | 284.0121,283.0060,256.0265,255.0167,227.0259,136.9870 | Diosmetin |
| 67 | 11.49 | C_17_H_14_O_7_ | 329.0667 | 329.0663 | -1.22 | 314.0202,298.9994,271.0084,227.0256,199.0355 | Jaceosidin |
| 68 | 11.51 | C_16_H_12_O_6_ | 299.0561 | 299.0551 | -3.34 | 284.0156,256.0251,227.0287,151.0043 | Chrysoeriol |
| 69 | 11.66 | C_16_H_12_O_7_ | 315.0510 | 315.0502 | -2.54 | 300.0052,151.0025,107.0169 | Tamarixetin |
| 70 | 11.73 | C_17_H_14_O_7_ | 329.0667 | 329.0669 | 0.61 | 314.0214,313.0112,298.9989,271.0083,243.0173,227.0258,199.0352 | Tricin |
| 71 | 13.47 | C_18_H_16_O_7_ | 343.0823 | 343.0818 | -1.46 | 328.03449,313.0129,285.0213,270.0023 | Eupatilin |
| 72 | 13.90 | C_16_H_12_O_5_ | 283.0612 | 283.0618 | 2.12 | 268.0221,239.0233,211.0328 | Wogonin |
| 73 | 13.93 | C_16_H_12_O_5_ | 283.0612 | 283.0610 | -0.71 | 268.0229,240.0319,239.036,211.0333,151.0045 | Acacetin |
| 74 | 21.95 | C_30_H_48_O_3_ | 455.3531 | 455.3530 | -0.22 | 407.2901 | Ursolic acid* |

* represents the use of the standards for comparison.
